# Untargeted Metabolomics: Biochemical Perturbations in Golestan Cohort Study Opium Users Inform Intervention Strategies

**DOI:** 10.3389/fnut.2020.584585

**Published:** 2020-12-22

**Authors:** Yuan-Yuan Li, Reza Ghanbari, Wimal Pathmasiri, Susan McRitchie, Hossein Poustchi, Amaneh Shayanrad, Gholamreza Roshandel, Arash Etemadi, Jonathan D. Pollock, Reza Malekzadeh, Susan C. J. Sumner

**Affiliations:** ^1^Department of Nutrition, Nutrition Research Institute, University of North Carolina at Chapel Hill, Chapel Hill, NC, United States; ^2^Digestive Oncology Research Center, Digestive Diseases Research Institute, Tehran University of Medical Science, Tehran, Iran; ^3^Golestan Research Center of Gastroenterology and Hepatology, Golestan University of Medical Sciences, Gorgan, Iran; ^4^Division of Cancer Epidemiology and Genetics, National Cancer Institute, Bethesda, MD, United States; ^5^Genetics, Epigenetics, and Developmental Neuroscience Branch, National Institute on Drug Abuse, Bethesda, MD, United States

**Keywords:** opium, opioid, exposome, metabolome, untargeted metabolomics, UPLC-MS, NMR

## Abstract

**Objective:** Over 50 million people worldwide are estimated to use opioids, of which ~30 million use opiates (opium and its derivatives). Use of opiates has been associated with a variety of adverse complications such as neurological and behavioral outcomes, addiction, cancers, diabetes, and cardiovascular disease. While it is well known that opiates exert their neurobiological effects through binding with mu, kappa, and delta receptors to exert analgesic and sedative effects, mechanistic links to other health effects are not well understood. Our study focuses on the identification of biochemical perturbations in Golestan Cohort Study (GCS) opium users.

**Methods:** We used untargeted metabolomics to evaluate the metabolic profiles of 218 opium users and 80 non-users participating in the GCS. Urine samples were obtained from adult (age 40–75) opium users living in the Golestan Province of Iran. Untargeted analysis of urine was conducted using a UPLC-Q-Exactive HFx Mass Spectrometry and a 700 MHz NMR Spectrometry.

**Results:** These GCS opium users had a significantly higher intake of tobacco and alcohol and a significantly decreased BMI compared with non-users. Metabolites derived from opium (codeine, morphine, and related glucuronides), nicotine, and curing or combustion of plant material were increased in opium users compared with non-users. Endogenous compounds which differentiated the opium users and non-users largely included vitamins and co-factors, metabolites involved in neurotransmission, Kreb's cycle, purine metabolism, central carbon metabolism, histone modification, and acetylation.

**Conclusions:** Our study reveals biochemical perturbations in GCS opium users that are important to the development of intervention strategies to mitigate against the development of adverse effects of substance abuse.

## Introduction

The use of opiates constitutes a major public health threat around the world and has been associated with neurological and behavioral outcomes, addiction, various cancers, diabetes, and cardiovascular disease. In 2017, the United Nations Office on Drugs and Crime (UNODC) estimated that there were 53.4 million opioid users worldwide (56% higher than 2016), of whom 29.2 million (50% higher than 2016) were opiate (opium and their derivatives) abusers (1). Opioids (both opiates and their synthetic analogs) are compounds that have similar pharmacologic effects as opium (2). Worldwide, opioids are the second most commonly used type of illicit drugs (only after cannabis) and are responsible for substantial morbidity and mortality, including two thirds of the 585,000 deaths from drug use disorder in 2017 (3). Analysis of health effects in a large prospective study suggested that opium users were at an 86% increased risk of death, due to higher rates of cancer and cardiovascular diseases (4).

Opium, a highly addictive drug, is the dried latex form the opium poppy plant (Papaver somniferum) and contains water, different types of sugars, several simple organic acids, and various alkaloids such as morphine (most prevalent and important alkaloid), codeine, thebaine, papaverine, and narcotine (5).

It is well known that opioids exert their neurobiological effects through binding with mu (μ), kappa (κ), and delta (δ) receptors (6), primarily in the central nervous system (CNS), and that they also have effects on these same receptors in the peripheral nervous system (PNS), gastrointestinal system, and immune system cells (7, 8). While the mechanisms for analgesic and sedative effects of opium consumption have been investigated, less is known regarding the mechanistic link to health effects (such as gastrointestinal cancers, cardiovascular disease, and diabetes) in opium users (9–12).

The identification of biochemical perturbations in opium users would significantly advance our understanding of mechanisms underlying disease phenotypes that have been linked with opium use. It is also expected that revealing metabolites and biochemical pathways perturbed by opium use would inform the development of biomarkers for monitoring addiction and withdrawal, as well as the potential to develop nutrition therapy strategies. In the recent decade, metabolomics has been used in opiate and opioid addiction research, mainly focused on understanding the biological mechanism underlying abuse, addiction and withdrawal symptoms, and many of these studies used experimental rodent models (13).

Our study was conducted to reveal biomarkers and gain insights into metabolic perturbations in opium users through analysis of urine obtained from adults (age 40–75) who participated in the GEMINI epidemiological study of 50,000 Iranians in the Golestan Province in Northeastern (4). Over 7,000 GEMINI participants self-reported daily use of opium (0.5 to 4.8 g; mean duration of ~13 years) through either smoking or orally consumption.

We used untargeted UPLC high resolution orbitrap mass spectrometry and NMR spectroscopy to reveal metabolites and biochemical pathway perturbations arising in a subset of GCS opium users compared with non-opium users. Our study reveals metabolic perturbations in GCS opium users that could inform the development of intervention strategies to mitigate against the development of adverse effects. To the best of our knowledge, our study was the first metabolomics investigation using both NMR and high resolution mass spectrometry to analyze human biospecimens collected from opium users and non-opium users, and to provide analysis of the biochemical perturbations that can inform nutritional intervention.

## Methods and Materials

### Study Population

We have previously published details of the GCS, a cohort of over 50,000 adults aged 40–75 living in Golestan Province, Northeast Iran (14). The GCS was approved by the ethics committees at Tehran University of Medical Sciences, the US National Cancer Institute (NCI), and the International Agency for Research on Cancer (IARC). Cohort participants provided non-fasted spot urine samples which were stored at −20°C until 2015 when they were transferred on dry ice to the NCI Biorepository and stored at −80°C. Aliquots were then shipped to UNC Chapel Hill. The study samples were selected from the GCS, to derive samples from 80 subjects who reported never using opium and from 218 opium users who were deemed high opium users based on the nokhods used per day. Since matched case-control designs are theoretically complex and may introduce bias, this exploratory study uses an unmatched case-control study design (15, 16). Details of the GCS study design and inclusion and exclusion criteria are found in Pourshams et al. (14). The 218 opium users are referred to as high opium users based on their reported nokhods consumption. All subjects selected for this study had only a history of opium use (no drugs other than nicotine, alcohol, and opium were used by participants in this sample). All samples were collected from residents of a small, local area in the northeast of Iran which has similarities in lifestyle, economics, and nutritional culture. While the amount of food consumed could be different among participants, as is expected due to differences in BMI, the types of intake are expected to be similar. Some of the subjects who contributed the 298 urine samples analyzed in this study reported one of the following chronic disease phenotypes (heart disease, hypertension, diabetes, jaundice, tuberculosis, obstructive pulmonary disease, or cancer). However, there was no significant difference (*p* = 0.36) in the total number of chronic diseases reported for the 218 opium users vs. the 80 non-users. We conducted a *post-hoc* power analysis for hypothesis testing using the *t*-test for two independent groups (n1 = 218, n2 = 80) using G^*^Power (17). We had 33.2% power to detect small effects (*d* = 0.2), 96.8% power to detect medium effects (*d* = 0.5), and 100% power to detect large effects (*d* = 0.8).

### Metabolomics Analysis via High Resolution Mass Spectrometry and NMR Spectroscopy

Details of the sample preparation, data acquisition, data preprocessing and metabolite identification and annotation are provided in the Supplementary Material section. Untargeted UPLCMS metabolomics data was acquired on a Vanquish UHPLC systems coupled with a Q Exactive™ HF-X Hybrid Quadrupole-Orbitrap™ Mass Spectrometer (UPLC-HR-MS; Thermo Fisher Scientific). Data was processed using Progenesis QI (Waters Corporation). Peaks detected by UPLC-HR-MS were identified or annotated. The evidence basis for metabolite identifications and annotations to the in-house library physical standards library (Ontology Level, OL), or Public Databases (PD), are detailed in the Supplementary Material. Untargeted NMR metabolomics data was acquired on an Avance III 700 MHz NMR (Bruker Corporation), and signals that differentiated the study groups were matched to metabolites using Chenomx NMR Suite 8.4 Professional software library.

### Hypothesis Testing

Statistical tests for the normalized peaks in the metabolomics profiles were conducted using a two-sided *t*-test with the Satterthwaite correction for unequal variances or the chi-square test. Statistical analyses were conducted using SAS 9.4 (SAS Institute Inc., Cary, NC). Nominal *p*-values are reported for the comparison of 218 opium users and the 80 non-user controls because this exploratory analysis was not powered for a specific hypothesis (18–20).

### Multivariate Statistics

Multivariate analysis was performed for the normalized data acquired by UPLC-HR-MS, or by NMR, using SIMCA 15.0 (Umetrics, Umeå, Sweden) to reduce the dimensionality and to enable the visualization of the differentiation of the study groups (SIMCA 15, Sartorius Stedim Data Analytics, AB, Umeå, Sweden) (21, 22). Unsupervised models were created using principal component analysis (PCA) and the scores plots were inspected to ensure that the QC pool samples were tightly clustered, and in the center of the study samples from which they were derived–a quality control method that is widely used in metabolomic studies (23). Orthogonal partial least squares discriminate analysis (OPLS-DA) was used to determine the variable influence on projection (VIP), for the normalized data from NMR and from UPLC-HR-MS, to define the signals important for differentiating the study groups. VIP ≥ 1.0 with a jack-knife confidence interval that did not include 0 were selected as important. The VIP statistic summarizes the importance of the bin/signal in differentiating the phenotypic groups (22). All models used a 7-fold cross-validation to assess the predictive variation of the model (Q2).

### Pathway Enrichment: Opium Users vs. Controls

Pathway enrichment was conducted using the Mummichog software (24) in Metaboanalyst 4.0 (25). All 7,714 features (m/z) remaining after filtering data were entered together with the *p*-value that was calculated for the comparison of opium users vs. controls. A *p*-value cut-off of 0.01 and a mass accuracy of 3 ppm were used for selecting significant features to match for all possible metabolites. All possible metabolites which were matched by m/z were searched in the human reference metabolic network (hsa_*m*_*fn*), and the null distribution of module activities were estimated by using 100 permutations of random lists drawn from the experimental reference feature list. The candidate pathways were based on the similarity of m/z.

### Biochemical Pathway Interpretation

#### Endogenous

Biochemical pathway interpretation was initiated with a classical approach of assessing the connection between analytes noted to significantly increase or decrease (VIP > 1 or *p* < 0.10 or |fold change| >2) between opium users and controls. The interpretations detailed in this manuscript include assessment of perturbations for vitamins, neurotransmitters, Kreb's cycle metabolism, and one carbon metabolism.

#### Exogenous

In addition, metabolites derived from opium, nicotine, and curing and combustion of plant material are described.

## Results

### Sample Characteristics

The subject characteristics (298 subjects total) for the 218 opium users and the 80 non-user controls are provided in Table 1. For the study samples evaluated herein, opium use was significantly associated with increased tobacco use (*p* = 3.9 × 10^−7^), increased alcohol use (*p* = 0.002), a lower body mass index (BMI, *p* = 4.3 × 10^−10^), the male gender (*p* = 0.008), and increased age at the time of enrollment (*p* = 0.039).

**Table 1:** Subject Characteristics for 218 Opium Users and 80 Non-users (Controls) from the GCS.

**Characteristic**	**Opium user (*n* = 218)**	**Non-user (*n* = 80)**	***p*-value^**2**^**
Age at enrollment years, mean (SD) [range]	49.8 (6.4) [39.7, 68.6]	48.1 (6.2) [39.7, 62.5]	**0.039**
Male (count, %)	172 (78.9%)	51 (63.8%)	**0.008**
Tobacco smoking status			**3.9** **×** **10**^**−7**^
Current smoker (count, %)	113 (51.8%)	19 (23.8%)	
Former smoker (count, %)	24 (11.0%)	3 (3.7%)	
Never smoker (count, %)	81 (37.2%)	58 (72.5%)	
Opium use, maximum nokhods per week (range)^1^	12.0 - 168.0	-	
Route of opium administration			
Inhalation	126 (57.8%)	-	
Ingestion	92 (42.2%)	-	
Body mass index, mean (SD)	23.7 (4.4)	28.2 (5.2)	**4.3** **×** **10**^**−10**^
History of alcohol use	54 (24.8)	7 (8.8)	**0.002**

1 *Nokhod is the local measurement for the amount of opium used, and is equivalent to ~0.2 grams (26)*.

2 *p-values < 0.05 are in bold text*.

### Metabolic Profiles

**Figure 1:**
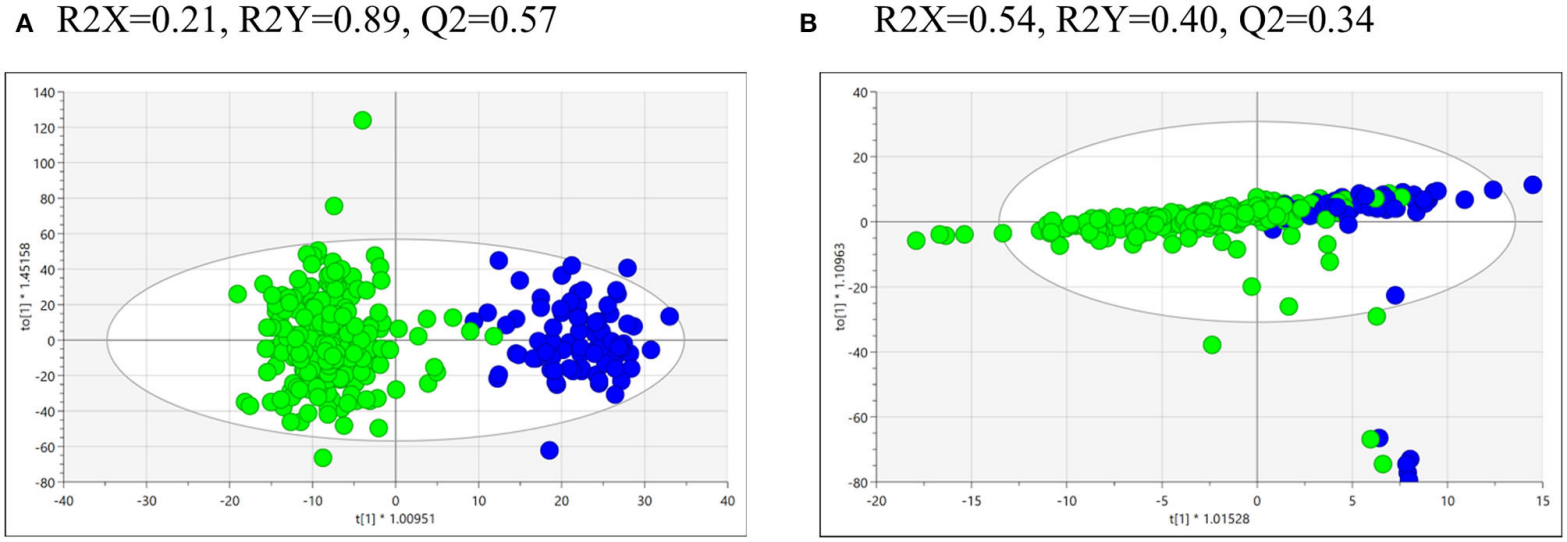
The OPLS-DA of the metabolomics data obtained by **(A)** UPLC-HR-MS analysis of urine samples from control (blue, right hand side) and GCS opium users (green, left hand side), or by **(B)** NMR analysis of urine samples from control (blue, left hand side) and GCS opium users (green, right hand side).

Statistics and multivariate analysis were used to compare the metabolomics profiles of the 218 opium users and the 80 controls. The supervised OPLS-DA of UPLC-HR-MS data for urine from the opium users vs. controls (Figure 1A) shows strong model statistics for outcome (R2Y = 0.89) and reproducibility (Q2 = 0.57, 7-fold cross validation). Over 4,866 signals met the criteria of VIP > 1, or *p* < 0.10, or absolute value of fold change >2 for differentiation of opium users and controls. Over 2,675 signals had *p* < 0.10, and over 2,099 signals had *p* < 0.05 for comparisons between opium users and controls (Table 2, Supplementary Table 1). The supervised OPLS-DA of NMR data for urine from opium user vs. control (Figure 1B) gives model statistics for outcome (R2Y = 0.40) and reproducibility (Q2 = 0.34, 7-fold cross validation). Over 120 bins met the criteria of VIP > 1, or *p* < 0.10, or absolute value of fold change >2 for differentiation of opium users and controls (Supplementary Table 2).

**Table 2:** Metabolites that most significantly differentiated GCS opium users from controls.

**Ontology level (OL)**	**Compound name (OL1, OL2a)**	**VIP**	*p*-value^*^	Fold Change^**^
OL1	Codeine	2.7	7.24E-40	22.6
OL1	Codeine	2.5	3.12E-35	42.9
OL1	Codeine-6-beta-D-glucuronide	2.6	6.32E-35	25.5
OL1	Morphine	2.3	2.62E-28	47.0
OL1	Morphine-3-beta-D-glucuronide	2.3	1.54E-27	117.4
OL1	Ferulate	2.1	4.18E-27	5.6
OL1	Morphine-6-beta-D-glucuronide	2.3	1.58E-25	139.1
OL1	N-Acetyl-S-(2-carbamoylethyl)-L-cysteine	2.0	4.16E-21	3.2
OL1	Pyridoxine	1.9	1.44E-16	1.8
OL1	Hydroxycotinine	1.7	6.88E-15	5.3
OL1	Cotinine	1.6	1.34E-10	3.8
OL1	Allothreonine	1.4	1.38E-09	1.4
OL1	Nicotine-N-oxide	1.4	2.93E-09	3.7
OL1	Nicotine	1.4	2.52E-08	4.5
OL1	Caffeic acid	1.0	2.02E-07	4.8
OL1	Phenethylamine	1.1	2.87E-07	1.6
OL1	Pantothenate	1.9	5.07E-07	−1.4
OL1	N-acetylcysteine	1.5	9.07E-07	1.4
OL1	N-Acetyl-S- (3-hydroxypropyl)-L-cysteine	1.5	1.07E-06	2.3
OL1	L-Tryptophan	1.5	2.08E-06	−1.4
OL1	Homovanillic acid	1.6	4.32E-06	−1.3
OL1	L-Tyrosine	1.4	9.11E-06	−1.4
OL1	DL-Leucine	1.7	1.15E-05	−1.6
OL1	3,5 dihydroxybenzyl alcohol	1.4	5.64E-05	−1.5
OL1	N-Methyl-L-glutamic acid	1.6	5.83E-05	−1.5
OL1	L-Isoleucine	1.4	1.09E-04	−1.4
OL1	N-Acetyl-DL-tryptophan	1.6	1.19E-04	−1.6
OL1	N-acetylglutamate	1.4	1.75E-04	−1.2
OL1	1-Methyl-L-histidine	0.8	1.01E-03	1.3
OL1	N-acetylasparagine	1.2	1.12E-03	−1.3
OL1	N-Acetyl-S- (3,4-dihydroxybutyl)-L-cysteine	1.3	1.36E-03	1.3
OL1	Biotin	1.2	3.60E-03	−1.5
OL1	Mevalonate	1.0	4.05E-03	−1.3
OL1	p-Methylhippuric acid	0.7	5.75E-03	1.7
OL1	10-hydroxydecanoic acid	1.1	6.11E-03	−1.3
OL1	Glucuronate	0.9	7.38E-03	1.3
OL1	L-carnitine	0.9	7.70E-03	−1.5
OL1	Pipecolate	1.1	8.81E-03	−1.8
OL1	N-acetylleucine	0.9	9.84E-03	−1.3
OL1	N,n-dimethyl-arginine	1.1	1.15E-02	1.1
OL1	Cytidine	1.3	1.26E-02	−1.1
OL1	Creatine	0.9	1.35E-02	−1.8
OL1	S-adenosylhomocysteine	1.2	1.60E-02	1.2
OL1	4-hydroxy-3-methoxyphenylglycol	1.1	1.83E-02	−1.4
OL1	Azelate	0.9	2.02E-02	−1.4
OL1	Anthranilate	0.8	2.11E-02	−1.5
OL1	Succinic acid	1.1	2.24E-02	−1.2
OL1	O-acetylcarnitine	0.9	2.82E-02	−1.7
OL1	N-acetylleucine	0.8	2.87E-02	−1.1
OL1	4-Hydroxyhippuric acid	0.7	2.87E-02	1.3
OL1	L-Methionine	1.0	3.06E-02	−1.2
OL1	2-aminophenol	0.7	3.19E-02	1.2
OL1	Suberate	0.9	3.30E-02	−1.3
OL1	3,4-Dihydroxybenzaldehyde	0.7	3.47E-02	1.3
OL1	Pyroglutamic acid	1.1	3.50E-02	−1.1
OL1	4-Pyridoxic acid	1.0	3.80E-02	−1.1
OL1	Raffinose	0.6	4.05E-02	−1.4
OL1	3-Hydroxy-3-methylglutaric acid	1.0	4.64E-02	−1.2
OL1	Cortisol	0.5	5.11E-02	1.3
OL1	Xanthurenate	0.9	5.78E-02	−1.1
OL1	N-Acetyl-D-galactosamine	1.0	6.48E-02	1.1
OL1	10-hydroxydecanoic acid	0.6	6.53E-02	−1.3
OL1	Trigonelline	0.6	6.68E-02	−1.2
OL1	Tryptamine	0.8	7.25E-02	−1.1
OL1	Betaine	0.7	7.42E-02	−1.3
OL1	Adenine	0.8	7.60E-02	−1.5
OL1	2,6-Diaminopimelic acid	0.5	8.23E-02	1.3
OL1	Adenosine	1.0	8.37E-02	1.1
OL1	N-methyltryptamine	0.6	8.50E-02	−1.4
OL1	N-acetylserine	0.7	9.36E-02	1.1
OL1	Hippuric acid	0.6	9.52E-02	−1.1
OL2A	DL-2-Aminoadipic acid	2.0	1.55E-26	3.8
OL2A	Codeine	2.2	1.23E-24	24.9
OL2A	5'-deoxyadenosine	2.1	3.29E-24	13.6
OL2A	Mono benzyl phthalate	1.7	1.50E-19	
OL2A	Indoleacetaldehyde	1.8	5.48E-19	3.5
OL2A	1-Aminocyclopropanecarboxylic acid	1.5	1.17E-12	1.5
OL2A	Indole-3-ethanol	2.0	8.64E-12	2.2
OL2A	4-Hydroxyhippuric acid	1.3	1.24E-11	8.2
OL2A	3-methoxytyramine	1.8	2.56E-11	1.5
OL2A	N-acetylputrescine	1.7	3.31E-10	1.3
OL2A	5-aminolevulinate	1.7	1.04E-09	1.8
OL2A	N-acetylalanine	1.6	6.33E-09	1.7
OL2A	Pyridoxal	1.4	1.12E-08	1.6
OL2A	Monoisopropyl phthalate	1.1	3.90E-08	16.2
OL2A	3-methylhistamine	1.2	8.37E-07	1.3
OL2A	Guanidineacetic acid	1.7	1.24E-06	−1.6
OL2A	Itaconate	1.4	2.48E-06	2.3
OL2A	N-Acetyl-S- (3,4-dihydroxybutyl)-L-cysteine	1.4	5.43E-06	1.4
OL2A	5-Methylcytosine hydrochloride	1.6	9.50E-06	−1.7
OL2A	3,4,5-trimethoxybenzaldehyde	0.8	1.20E-05	2.7
OL2A	Taurine	1.2	3.81E-05	1.6
OL2A	N-acetylphenylalanine	1.6	1.55E-04	−1.9
OL2A	Mono (2-ethyl-5-hydroxyhexyl) phthalate	1.6	1.06E-03	−1.9
OL2A	L-Proline	1.1	1.51E-03	1.8
OL2A	Deoxyadenosine	1.2	2.60E-03	−1.2
OL2A	Homoveratric acid	1.1	2.86E-03	−1.2
OL2A	Threonine	0.9	3.45E-03	−1.5
OL2A	3-(carbamoylamino)propanoic acid	1.3	3.75E-03	−1.3
OL2A	6-carboxyhexanoate	1.0	3.88E-03	−1.3
OL2A	Methyglutarate	1.1	4.34E-03	−1.2
OL2A	Anserine	0.9	5.25E-03	−2.1
OL2A	4-acetamidobutanoic acid	1.3	5.42E-03	−1.1
OL2A	Nicotinamide	0.5	6.91E-03	1.7
OL2A	Aniline-2-sulfonate	1.1	8.76E-03	−1.4
OL2A	Methyl galactoside	1.2	1.36E-02	−2.3
OL2A	Kynurenine	0.9	1.38E-02	−1.3
OL2A	1-methyladenosine	1.0	1.98E-02	−1.5
OL2A	N-acetyl-S- (3,4-dihydroxybutyl)-L-cysteine	0.5	2.39E-02	3.1
OL2A	N-acetylphenylalanine	0.7	2.40E-02	1.6
OL2A	3,4,5-trimethoxybenzaldehyde	0.9	2.40E-02	−1.6
OL2A	Cytidine	0.7	2.89E-02	1.2
OL2A	5-hydroxytryptophan	1.2	2.91E-02	−1.2
OL2A	N-acetylalanine	1.0	2.92E-02	−1.1
OL2A	N-acetylproline	1.0	3.05E-02	−1.2
OL2A	Uridine	0.9	3.48E-02	−1.2
OL2A	Creatinine	1.2	3.84E-02	−1.1
OL2A	Hydrocinnamic acid	0.5	4.85E-02	1.9
OL2A	O-acetylcarnitine	0.7	4.86E-02	1.2
OL2A	Monoethyl phthalate	1.0	4.92E-02	−1.2
OL2A	Mannose	0.9	5.09E-02	−4.1
OL2A	Homoveratric acid	0.8	7.97E-02	−1.4
OL2A	Estradiol-17alpha	0.5	8.07E-02	1.4
OL2A	Sebacate	1.0	9.00E-02	−1.2
OL2A	6-hydroxypyridine-3-carboxylic acid	1.0	9.38E-02	−1.2

*Additional metabolites that met the criteria of VIP ≥1, p < 0.10, or foldchange ≥2 are reported in Supplementary Tables*.

* *t-test with Satterthwaite correction for unequal variances*.

** *Positive fold change - mean Opium User > mean Non-Opium User*.

### Pathway Enrichment Based on Metabolic Profiles

**Figure 2:**
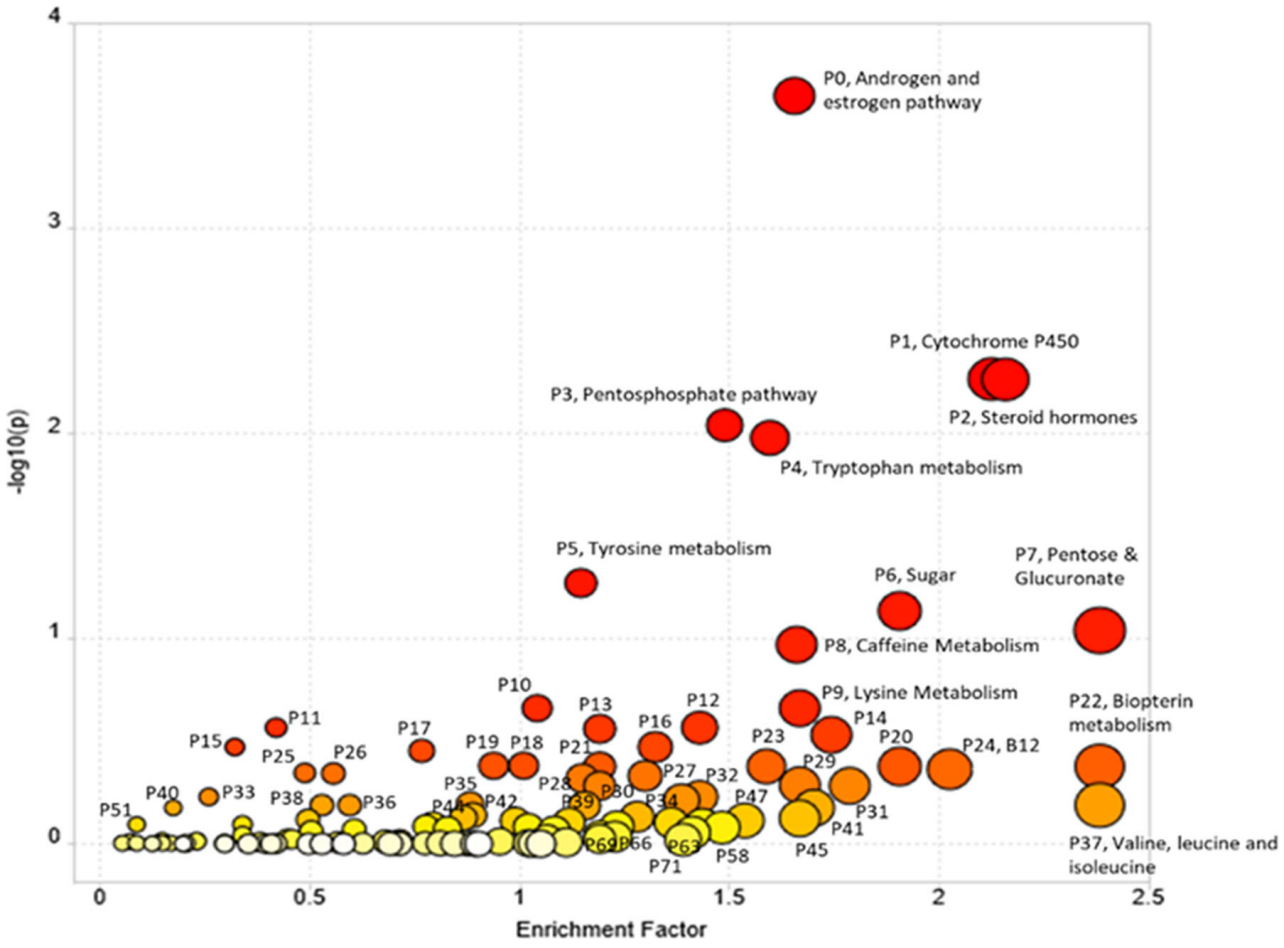
The Pathway Enrichment vs. –log10 (p) with *p* < 0.01 cutoff for comparison of GCS opium users and controls.

Pathway enrichment using Mummichog resulted in 15,838 annotations to compounds or adducts (resulting in 1,565 unique putative compound IDs). A cut-off of *p* < 0.01 for comparison of opium users vs. controls resulted in 2,189 significant features that were selected for pathway enrichment analysis. The plot of pathway enrichment factor vs. –log10 (p) is shown in Figure 2, and pathways deemed significant by both the fisher's test and gamma distribution are labeled. The top ten enriched pathways are listed in Table 3 (for the extended list of pathways, see Supplementary Table 3), and the signals identified or annotated as significantly different between opium users and controls are provided in Supplementary Table 1. Hundreds of signals were annotated via the Mummichog pathway enrichment. Signals associated with these enriched pathways, that were significantly different between opium users and non-opium users, and that were identified or annotated using our in-house physical standards library and public databases are described.

**Table 3:** Top 10 candidate pathways, based on the similarity of m/z using Mummichog, that differentiated opium users from controls.

**Pathway name**	**^a^Pathway total**	**^b^Hits.total**	**^c^Hits.sig**	**^d^Gamma**	**^e^Pathway Number**
Androgen and estrogen biosynthesis and metabolism	95	69	66	0.002824	P0
Drug metabolism–cytochrome P450	53	51	48	0.002989	P1
C21-steroid hormone biosynthesis and metabolism	112	77	70	0.003025	P2
Pentose phosphate pathway	37	34	33	0.003062	P3
Tryptophan metabolism	94	69	63	0.003068	P4
Tyrosine metabolism	160	88	77	0.003865	P5
Fructose and mannose metabolism	33	25	23	0.006684	P6
Pentose and Glucuronate Interconversions	15	12	12	0.007655	P7
Caffeine metabolism	11	11	11	0.009114	P8
Lysine metabolism	52	30	26	0.014088	P9

a *Pathway total indicates the overall number of metabolites that are included in a specific pathway*.

b *Hits.total indicates the number of measured signals that are matched (m/z error <3 ppm) with the metabolites included in the pathway*.

c *Hits.sig indicates the number of matched signals that were significantly changed between phenotypic groups*.

d *Gamma is an adjusted Fisher's p-value (null distribution) calculated after permutations to determine the significance of the enriched pathway in Mummichog/ Metaboanalyst (24, 25)*.

e *Pathway Number listed in the table corresponds to that in Figure 2*.

#### P0 and P2: Androgen and Estrogen Biosynthesis and Metabolism, and Hormone and Steroid Metabolism

Mevalonate (OL1, *p* = 4.1E-3) an important precursor for biosynthesis of steroids (OL1, *p* = 4.1E-3), cortisol (OL1, *p* = 0.051) and estradiol (OL2a, *p* = 0.081) were increased in opium users compared with controls. Over 30 steroid hormones and related derivates were significantly differentiated (*p* < 0.05) between the opium users and controls which were annotated by matching to public databases (PD levels, Supplementary Table 1). Metabolites matching by mass and experimental MS/MS (PDa) include: 5-α-androsterone, 11-β-hydroxyandrosterone, 5-α-pregnane-3,20-dione, and 4-androsten-17-β-ol-3-one glucosiduronate.

#### P1 (and P21): Drug (and Xenobiotics) Metabolism–Cytochrome P450

Uridine 5′-diphospho-glucuronosyltransferase (UGT), and metabolites derived from opium and nicotine (described below) were significantly different between opium users and controls (Supplementary Table 1).

#### P4 and P5: Tryptophan and Tyrosine Metabolism

**Figure 3:**
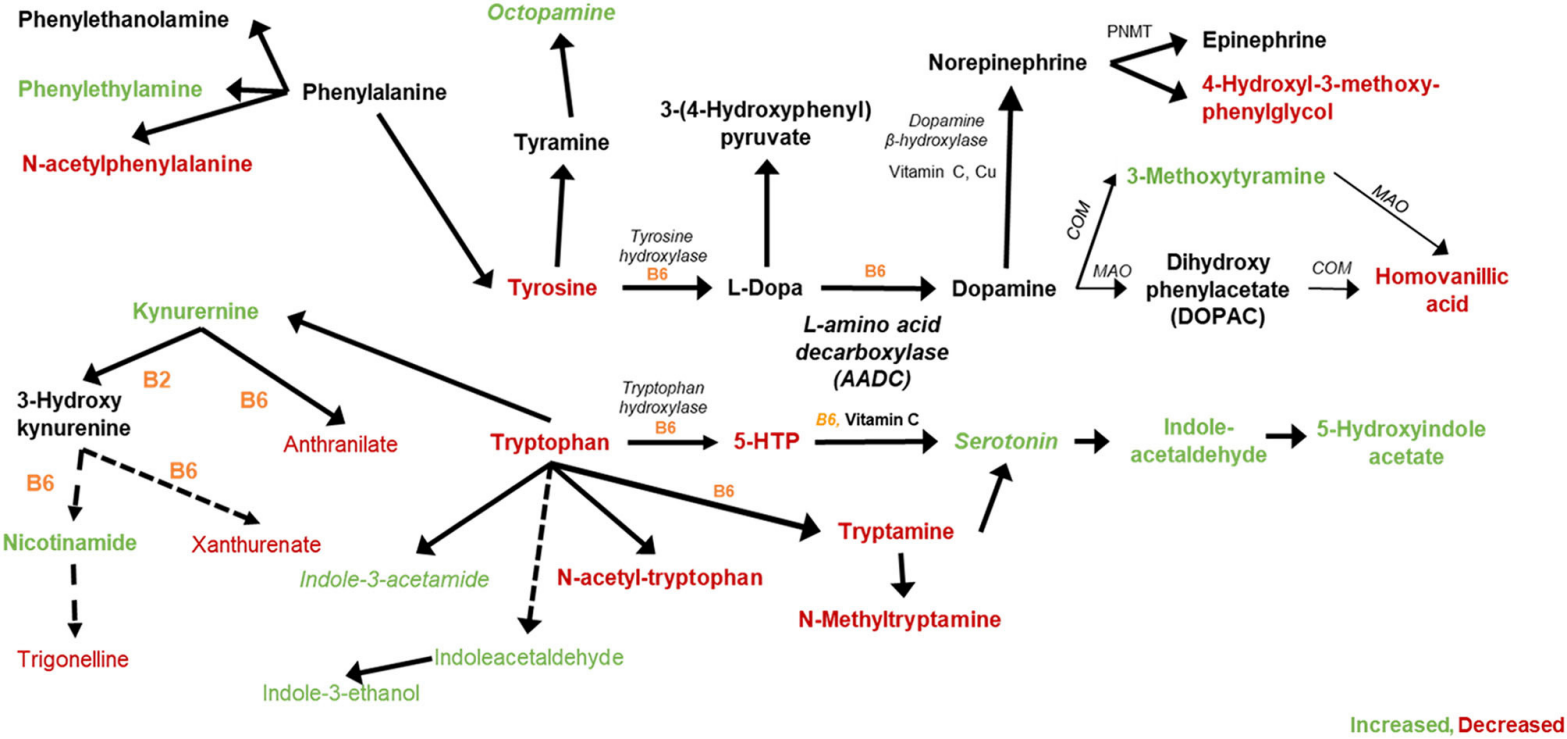
Tryptophan, Tyrosine, and related metabolites that were increased (green) or decreased (red) in GCS opium users compared with controls.

Significant pathway perturbations were detected between opium users and non-users, which are consistent with signals identified and annotated in our study that are involved in tryptophan and tyrosine metabolism (detailed in Figure 3).

#### P3, P6, P7: Sugar Metabolism, Pentose Phosphate Pathway, and Pentose and Glucuronate Interconversions

**Figure 4:**
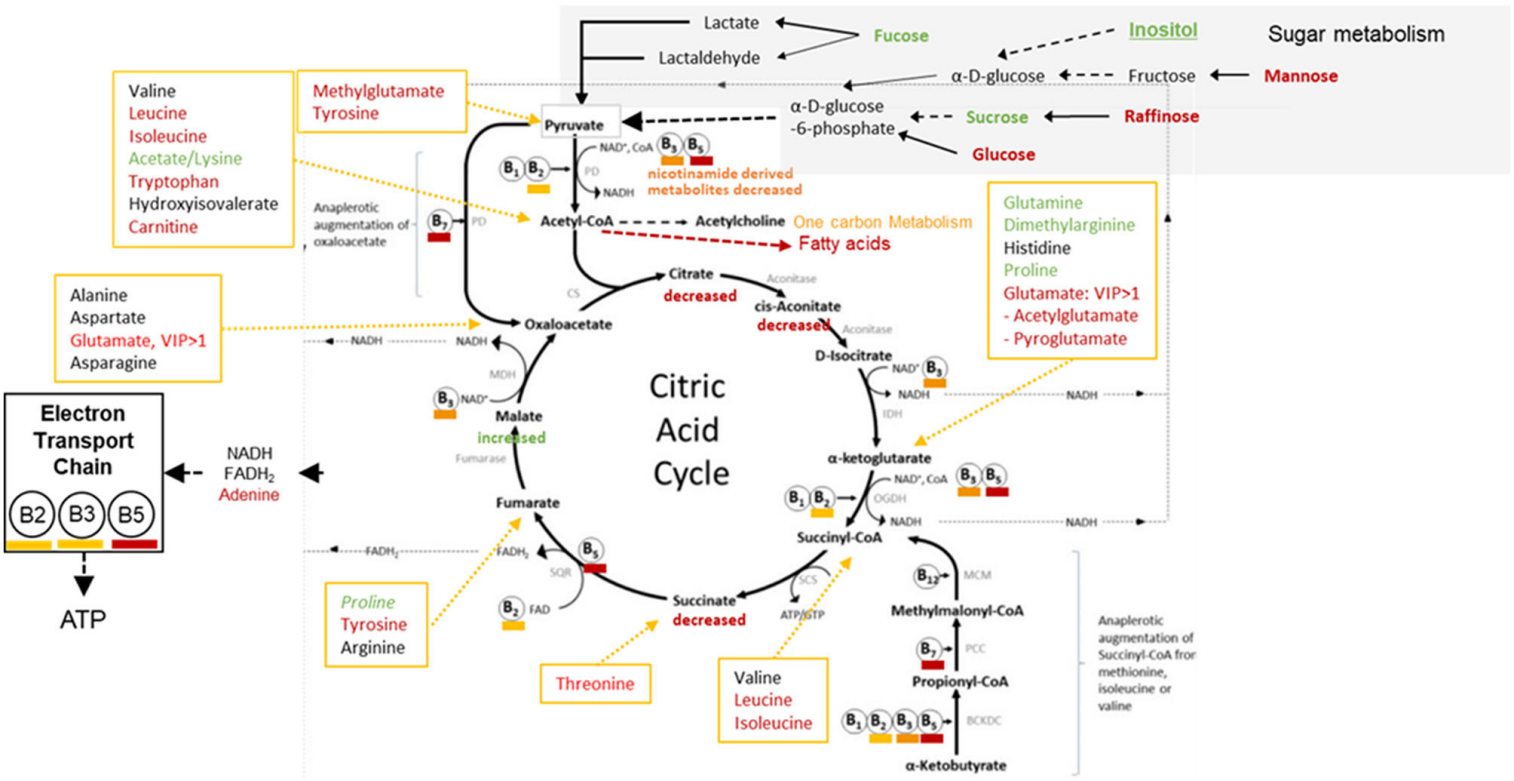
GCS Opium Users: Perturbing the Pyruvate Dehydrogenase Complex, Krebs Cycle, and the Impact of the Electron Transport Chain [extensively modified, (27)].

Significant perturbations were detected between opium users and controls for metabolites involved in sugar metabolism (Figure 4). Key compounds (Supplementary Tables 1, 2) that significantly differentiated the opium users and controls and could contribute to the P3, P6, and P7 pathway perturbations were identified/annotated by UPLC-HR-MS (succinate, OL1; raffinose, OL1; mannose, OL2a; glucosamine, OL2b) and by NMR (fucose, citrate).

### Endogenous and Exogenous Biochemical Perturbations

Over 200 signals that differentiated opium users and controls (VIP >1, or *p* < 0.10, or absolute value of fold change >2) were matched using the in-house physical standards library (UPLC-HR-MS, 177, Supplementary Table 1) or Chenomx library (NMR, 87, Supplementary Table 2). These metabolites are classified as derived from opium use, tobacco exposure, perturbations in neurotransmitter metabolism, Krebs cycle metabolism, one carbon metabolism, glucogenesis, lipid metabolism, and vitamin metabolism or utilization.

#### Exogenous Exposures

##### Opium Related Metabolites

The *p-*value was calculated for comparison of the high intensity signal in individuals reporting opium use, and background signals determined for the controls reporting no-opium use. Signals matched to codeine (OL1, *p* = 7.2E-40,), codeine 6-beta-glucuronide (OL1, *p* = 6.3E-35), morphine (OL1, *p* = 2.6E-28), morphine-3-beta-glucuronide (OL1, *p* = 1.5E-27), and morphine-6-beta-glucuronide (OL1, *p* = 1.6E-25) were drastically higher in opium users than for individuals reporting no opium use. Additional opium related analytes that were annotated included dihydromorphine (OL2b), naloxone-3-beta-D-glucuronide (OL2b), noscapine (PDa), cephalotaxine (PDa), and hydrocotarnine (PDa).

##### Tobacco Related Metabolites

Consistent with the subject characteristics, tobacco and tobacco related metabolites were significantly increased in opium users vs. controls. This included: nicotine (OL1, *p* = 2.52E-8, fold change >4), and metabolites derived from nicotine [nicotine-N-oxide (OL1, *p* < 2.93E-9, fold change >3), trans-3'-hydroxycotinine (OL2b), cotinine (OL1, *p* = 1.34E-10, fold change >3), and hydroxycotinine (OL1, *p* = 6.88E-15, fold change >5)]. A signal matching anatabine, a nicotine related alkaloid, was also significantly increased in opium users (OL2b, *p* = 1.90E-20, fold change >38) compared with controls. Additional tobacco related analytes that were annotated included anatabine (OL2b) and megastigmatrienone (PDa), dihydroactinidiolide (PDa), and 6-hydroxypseudooxynicotine (PDa).

### Metabolites Related to Plant Combustion

Metabolites that were significantly increased in opium users over controls have previously been associated with tobacco use. These include N-Acetyl-S- (3,4-dihydroxybutyl)-L-cysteine (OL1, *p* = 1.36E-3, fold change >5), N-Acetyl-S- (3-hydroxypropyl)-L-cysteine (OL1, *p* = 1.07E-6, fold change >2), and N-Acetyl-S-(2-carbamoylethyl)-L-cysteine (28) (OL1, *p* = 4.16E-21, fold change >3). These compounds are known urinary metabolites of parent compounds (butadiene, acrylamide, and acrolein) that could be formed during curing or on combustion of plant material (28–30).

### Metabolites of Phthalate Exposure

Monoisopropyl phthalate (OL2a, *p* < 0.0001, fold change>16) was dramatical increased in opium users compared with non-opium users. Other phthalates were also significantly different, including monomethyl phthalate (OL2b, *p* = 0.028, fold change = 1.2), mono-2-ethyl-5-hydroxyhexyl phthalate (OL2a, *p* < 0.001, -fold change = 1.9), and monoethyl phthalate (OL2a, *p* = 0.049, -fold change = 1.2).

#### Endogenous Metabolites

##### Neurotransmitter Pathway

Metabolites that increased (green) or decreased (red) in opium users vs. non-opium users that are associated with the metabolism of tryptophan and tyrosine are shown in Figure 3. Metabolites matching (OL1) to the in-house library that were significantly different (*p* < 0.10) between opium users and controls include tryptophan (*p* = 2.1E-6), tyrosine (*p* = 9.1E-6), xanthurenate (*p* = 0.058), trigonelline (*p* = 0.067), tryptamine (*p* = 0.073), anthranilate (*p* = 0.021), 4-hydroxy-3-methoxyphenyglycol (*p* = 0.018), homovanillic acid (*p* = 4.3E-6), and phenethylamine (*p* = 2.8E-7). Additional matches by RT and Exact Mass (OL2a) include kynurenine (*p* < 0.014), indoleacetaldehyde (*p* = 5.5E-19), indole-3-ethanol (*p* = 8.6E-12), 3-methoxytyramine (*p* = 2.6E-11), N-acetylphenylalanine (*p* = 1.6E-4), and 6-hydroxypyridine-3-carboxylic acid (6-hydroxynicotinic acid; *p* = 0.094). Signals that matched to standards in the in-house library by exact mass and MS/MS fragmentation (OL2b) included octopamine (*p* = 7.4E-8), serotonin (*p* = 7.6E-13), indole-3-acetamide (*p* = 4.8E-3), 5-hydroxyindoleacetate (*p* < 0.016). 1-Aminocyclopropane-1-carboxylic acid (OL2a, *p* = 1.2E-12), a partial agonist of the glutamate receptor, NMDA, was increased in opium users, as was N-acetyl glutamate (OL1, *p* = 1.8E-4), N-methyl glutamate (OL1, *p* = 5.8E-5), and pyroglutamate (OL1, *p* = 0.035). Azelate, known as an inhibitor of tyrosinase (the enzyme that converts tyrosine to L-DOPA in melanocytes and is an alternative pathway for converting tyrosine to L-dopa in the nervous system), was decreased (OL1, *p* = 0.020) in opium users.

##### Vitamins and Co-factors

Vitamins and related metabolites that were perturbed when comparing opium users with controls include vitamin B5 (pantothenate, OL1, *p* = 5.1E-7), vitamin B7 (biotin, OL1, *p* = 3.6E-3), Vitamin B3 (nicotinamide, OL2a, *p* = 6.9E-3), and the Vitamin B6 family of pyridoxine (OL1, *p* = 1.4E-16), pyridoxal (OL2a, *p* = 1.1E-8), 4-pyridoxic acid (OL1, *p* = 0.038). Riboflavin was 2-fold lower in opium users than in non-opium users.

##### Sugar Metabolism, Kreb's Cycle, and the Electron Transport Chain

Perturbations in Sugar metabolism, Kreb's Cycle metabolism, and the influence on the Electron Transport Chain are shown in Figure 4. Signals matched to glucose, raffinose, mannose, fucose, sucrose, and inositol were perturbed (*p* < 0.05) in opium users compared with controls. Perturbations in sugar metabolism, together with disruption in vitamin utilization or metabolism, could influence the production of acetyl-CoA, and subsequently disrupt metabolic pathways that depend on entrance of acetyl-CoA (e.g., metabolism of fatty acids, one carbon metabolism, Krebs cycle).

Metabolites related to Kreb's cycle (Figure 4) that were perturbed (*p* < 0.05) between opium users and non-users include citrate, aconitate, succinate, itaconate, malate, amino acids (e.g., glutamine, dimethylglutamine, proline, leucine, isoleucine, threonine, tyrosine). It is feasible that perturbations in sugar metabolism, together with differences in the utilization or metabolism of vitamins (B2, B3, B5, B7) and the resultant Kreb's cycle disruption, could decrease the production of NADH and FADH2.

The decrease in methylcytosine (*p* = 9.5E-6), cytidine (*p* = 2.9E-2), and uridine (3.5E-2), and the related decrease in adenine (*p* = 0.076), together with perturbations in vitamin metabolism could significantly impact the production of ATP via the Electron Transport Chain.

##### Central One Carbon Metabolism

**Figure 5:**
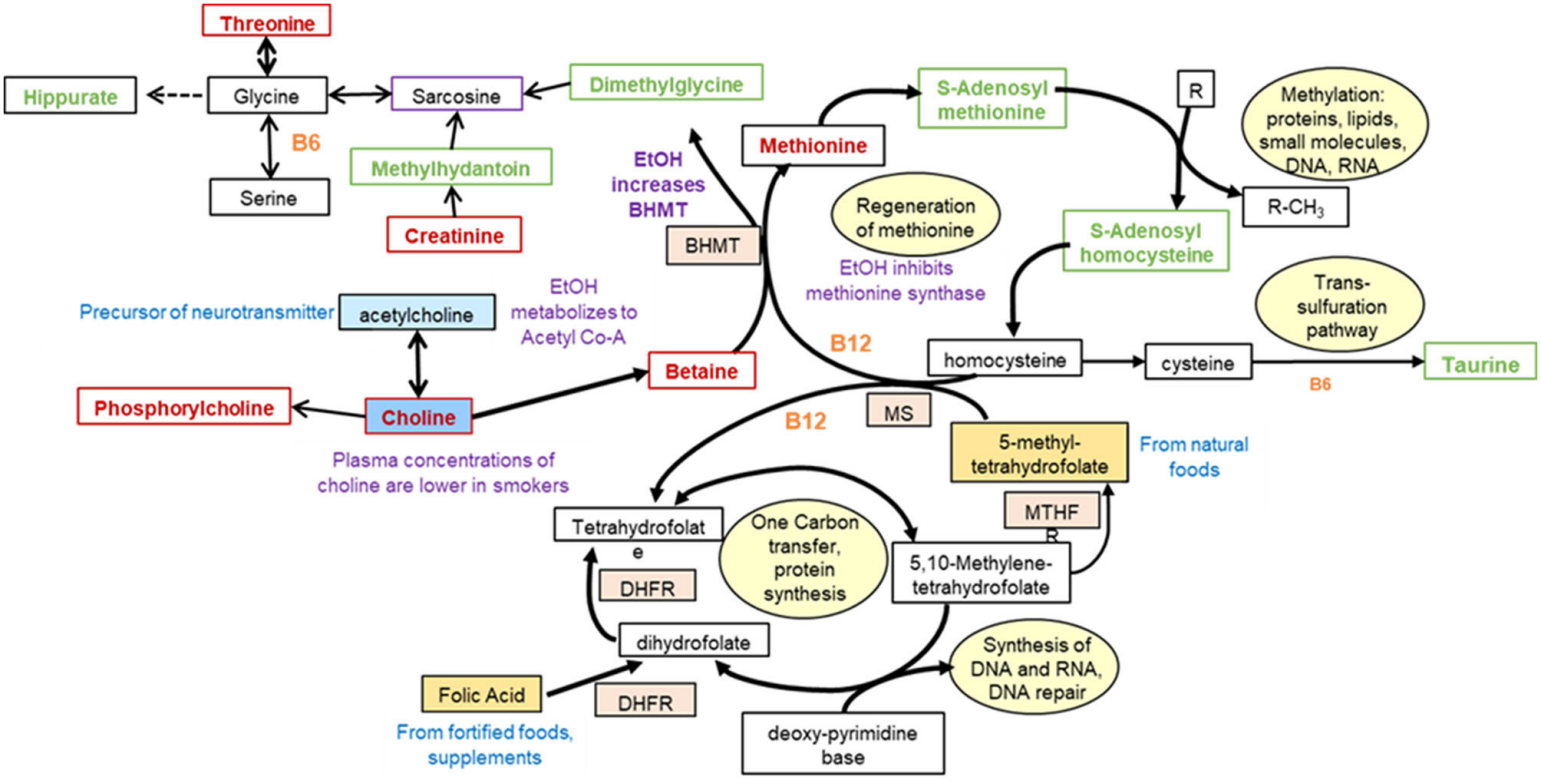
Perturbations in one carbon metabolism in GCS opium users [modified from (31)].

Perturbations in one carbon metabolism are shown in Figure 5. The decrease (*p* < 0.05) in choline and phosphorylcholine could be associated with decrease in acetyl-CoA production as a result of decreased utilization and metabolism of vitamins, and a decrease in glucose in opium users compared with controls. An increased demand for methylation of proteins, lipids, or small molecules could shift metabolism toward the increase in S-adenosyl methionine and S-adenosylhomocysteine (OL1, *p* = 0.016), decreasing methionine (OL1, *p* = 0.013). Perturbations in hippurate (OL1, *p* = 0.095), methyl hippurate (*p* = 5.6E-3), and 4-hyroxyhippurate (OL1, *p* = 0.049) may occur through interruption in the production through glycine. Increased hippuric acid has been associated with tyrosinemia, an error in metabolism that prevents effective breakdown of tyrosine and could be associated with liver and kidney disease. Taurine (OL2a, *p* = 3.8E-5), an essential sulfur containing amino acid which can serve as a neurotransmitter, was increased in the urine of opium users. In addition, perturbation of metabolites containing a pterin moiety, including biopterin (PDa), tetrahydro-L-biopterin (PDa), and neopterin (PDa), could be associated with the shift of folate metabolism.

##### Methylated Amino Acids

Some methylated amino acids were increased in opium users compared with controls, including N,N-Dimethyl arginine (OL1, *p* = 0.012), 1-Methyl-L-histidine (OL1, *p* = 1.0E-3), while N-Methyl-L-glutamic acid (OL1, *p* = 5.8E-5), methyltryptamine (OL1, *p* = 8.5E-2), methylglutarate (OL2a, *p* = 4.3E-3), methyladenosine (OL2a, *p* = 0.020), and methylcytosine (OL1, *p* = 9.5E-6) were decreased.

##### Acetylated Metabolites

Opium users had increased acetylated metabolites including N-acetyl cystine (OL1, *p* = 9.1E-7), N-Acetyl-D-galactosamine (OL1, 0.065), methylhistamine (OL2a, *p* = 8,4E-7), and N-acetylserine (OL1, *p* = 0.094). N-acetyl alanine, (OL2a, *p* = 6.3E-9), N-acetyl-asparagine (OL1, *p* = 1.1E-3), N-acetyl-glutamate (OL1, *p* = 1.8E-4), acetyleucine (OL1, *p* = 0.029), and N-acetyl-tryptophan (OL1, *p* = 1.2E-4) were decreased in opium users. Over 10 acylated amino acids (OL2b or PDa) that differentiated (*p* < 0.1) opium users and non-users were annotated (Supplementary Table 1), including those that area classified as neurotransmitters, such as N-acetyl-phenylalanine and N-acetyl-L-glutamic acid and those polyamines that function as modulators of neurotransmission, including N-acetylcadaverine, N1-acetylspermidine, and N-acetyl putrescine.

##### Heme Synthesis

The metabolism of aminolevulinic acid (ALA) is the first step in the biochemical pathway resulting in heme synthesis. Higher levels of ALA (OL1, *p* = 1.0E-9) in opium users compared with controls may indicate underutilization for the synthesis of heme.

##### Fatty Acids

Suberate (OL1, *p* = 0.022), sebacate (OL2a, *p* = 0.090), 10-hydroxydecanoic acid (OL1, *p* = 0.006), and carboxyhexanoate (Ol2a, *p* = 3.9E-3) were decreased in opium users. Over 15 compounds were annotated that also revealed perturbations in fatty acid metabolism.

##### Carnitines

*C*arnitine (OL1, *p* = 7,7E-3) and acetyl carnitine (OL1, *p* = 0.028) were decreased in opium users. Other signals annotated (PDa) as carnitine derivatives, including butyryl-L-carnitine, hexanoylcarnitine, decanoyl-L-carnitine, and isobutyryl carnitine, were decreased in opium users.

##### Lysine Metabolism

Diaminopimelic acid (OL1, *p* = 0.082) is a lysine-like amino acid derivative that is a key component of the bacterial cell wall, and may increase in urine due to breakdown of gram negative gut microbes. Pipecolic acid (OL1, *p* = 8.8E-3) is a metabolite of lysine and is decreased in opium users. Pipecolate has been associated with B6 pyridoxine-dependent seizures (32, 33).

## Discussion

This metabolomics investigation of a subset of urine samples from the GCS reveal significant biochemical perturbations in GCS opium users compared to non-opium users (Figures 2–5). These GCS opium users also had a significantly higher use of alcohol and tobacco compared with non-opium users. The use of alcohol or tobacco concurrently with drugs of abuse has been documented for GCS participants, as well as in other cohort investigation drugs of abuse (34, 35).

Because the use of illicit drugs is often accompanied by alcohol and tobacco use, this sample from the GCS is ideal for the assessment of biochemical perturbations that arise from the common concurrent exposures of alcohol and tobacco together with opium. Understanding metabolic perturbations that occur simultaneously from multiple common exposures is necessary to inform intervention strategies. As expected, our analysis demonstrated that the GCS opium users had the presence of metabolites that are derived from opium, and metabolites derived from tobacco were at levels significantly higher than non-users. In addition, N-acetyl cysteine conjugates that could be derived during the metabolism of known chemical carcinogens (e.g., acrylamide, acrylonitrile) are significantly increased levels in GCS opium users compared with non-opium users. Early studies have shown increased levels of these metabolites in urine from tobacco users (28, 30, 36, 37) and have demonstrated the formation of the parent chemical carcinogens from combustion of plant matter (28, 38–40). It is possible that the increased rates of cancer among GCS opium users is in part related to the presence of these chemical carcinogens (41–43). Urinary metabolites that are derived from phthalates were also detected at higher levels in the GCS opium users compared with non-users. It is possible that opium users are exposed to higher concentrations of some phthalates through plastic tubing used in devices for opium delivery (e.g., hookah pipes). Phthalates have been associated with a wide range of health outcomes, including diabetes (44), cancers (45, 46), cardiovascular disease (47), and cognition (48).

Endogenous compounds which differentiated the opium users and non-users largely included vitamins and co-factors, and metabolites involved in neurotransmission, Kreb's cycle, purine metabolism, central carbon metabolism, histone modification, and acetylation (Figures 2–5). The perturbations in host metabolism are highly consistent with the published results from experimental animals that were exposed to opiates and opioids (13, 49–52).

Exposures to alcohol, tobacco, and illicit drugs are known to impact the absorption and utilization of vitamins and minerals (53). A decreased BMI in these GCS opium users compared with non-users is consistent with historical literature indicating nutritional deficiencies associated with use of tobacco and drugs (54) and could be related to perturbations in metabolites involved in heme synthesis.

B-vitamins are required to convert pyruvate to acetyl-coA, which is then utilized on numerous biochemical pathways (e.g., Kreb's cycle, fatty acids, one carbon metabolism) (Figures 4, 5). The significant reduction in pantothenate (Vitamin B5), which has a wide dietary availability, suggest that GCS opium users have a lower food consumption that non-users, yet it is also possible that GCS opium users have impaired absorption of the B5 through alterations in the gut microbiome (52).

Many of the vitamins that were perturbed in this study and whose reduced levels have been associated with use of tobacco, drugs, and alcohol are involved in production of neurotransmitters and in the production of ATP (Figures 4, 5). Perturbations in neurotransmitters and decreased ATP production could be related to a wide range of disease outcomes for GCS opium users including cancer (35, 55), heart disease (4, 56), and cognition (57).

Chronic exposure to opioids is associated with increased global H3 histone acetylation in the mesolimbic dopamine system of rodents and in the striatum in post-mortem heroin users, with histone acetylation occurring on the lysine tails of H3K9, H3K14, H3K18, H3K27 (58). Histone acetylation is associated with an open chromatin conformation to enable increased gene transcription. Acetyl-CoA is the major substrate for acetylation of histones. Alterations in the amounts of precursors, synthesis, transport, enzymatic activity of histone acetylases could affect the amount of histone acetylation. In addition, mutations in histones could affect the amount of histone acetylation. At the same time alterations in DNA sequence could affect the response to acetylation by preventing change in conformation following acetylation. A cascade of events that start with decreased vitamins and cofactors, decreased acetyl co-A, and perturbations in one carbon metabolism can influence DNA methylation, and histone modification (59), which have been associated with exposure to tobacco, alcohol or opium.

Our results show that GCS opium users have disruptions in vitamin metabolism required for the production of Acetyl-CoA, the TCA cycle, and one carbon metabolism. This cascade may explain the observation of a decrease in in H3K9 dimethylation (H3K9me2) in the nucleus accumbens and the central amygdala of the mouse. Alterations in the synthesis or increased availability of alpha-ketoglutarate, a required cofactor for KDM histone demethylases could lead to demethylation of H3K9me2 (60). For decades, drug addiction research has focused on the discovery of druggable targets to develop therapeutics to prevent addiction and to mitigate against withdrawal and relapse. The results of our study clearly demonstrates the importance of considering multiple exposures and multiple targets in the development of a therapeutic to mitigate against adverse effects. Over 50 years ago, Dole and Nyswander described the acquisition of addiction as being initiated through a metabolic imbalance.

Validation of the discovered metabolic perturbations that resulted in decreased vitamins and vitamin-like compounds, fatty acids, carnitines, and amino acids (e.g., tryptophan) in opium users could lead to the development of a nutrient cocktail to test in clinical settings for efficacy to mitigate symptoms associated with opioid use. A clinical trial conducted with a combination cocktail of nutrients and vitamins, together with drug candidates that target opioids may be the most successful approach to mitigate against addiction and the adverse health consequences associated with the use of drugs of addiction. Limitations to this study include that the non-fasted spot urine were not collected at the same time of day for all individuals, the sample size for opium users and non-opium users were not the same and were not matched on all variables which could be confounders (e.g., age, sex, BMI). These factors could have an influence on the results. The biological mechanisms specific to codeine or to morphine will be the subject of future analysis. This study should be replicated in a second cohort.

## Data Availability Statement

This data is available at the NIH Common Fund's National Metabolomics Data Repository (NMDR) website, the Metabolomics Workbench, https://www.metabolomicsworkbench.org Project ID: PR001038 DOI: http://dx.doi.org/10.21228/M8HX30.

## Ethics Statement

The studies involving human participants were reviewed and approved by The Central Institutional Review Board for the National Cancer Institute, NIH, 401 N. Washington Street, Ste. 700 Rockville, MD 20850, USA. The patients/participants provided their written informed consent to participate in this study.

## Disclosure

The views and opinions expressed in this manuscript are those of the authors only and do not necessarily represent the views, official policy or position of the U.S. Department of Health and Human Services or any of its affiliated institutions or agencies.

## Author Contributions

Y-YL, RG, and WP: sample preparation, mass spectrometry and NMR data acquisition, multivariate analysis, identification and annotations of signals, pathway analysis, and manuscript preparation. SM: statistical analysis of subject characteristic data and metabolomics data, provided text, figures, and tables for the methods, results, and Supplementary Material. HP: input into the design of Golestan cohort study, data analysis, and training of the interviewers. AS: contributed to the sampling as well as filling out the questionnaires. GR: design of questionnaire and analysis the Golestan cohort data. AE: intellectual input into the design of the experiments, and preparation of the methods section. JP: intellectual input into the design of the experiments as well as this manuscript. RM: intellectual input for the design of the experiments as well as accomplishment of Golestan cohort study and supervised all data and biospecimen collection, and provided review and confirmation of the final version of the manuscript. SS: intellectual input for the design of the metabolomics experiment, interpretation of the metabolite and pathway perturbations, prepared text for all sections of the manuscript and Supplementary Material, prepared figures. All authors contributed to the article and approved the submitted version.

## Conflict of Interest

The authors declare that the research was conducted in the absence of any commercial or financial relationships that could be construed as a potential conflict of interest.
