# Supplementary material for: Untargeted Metabolomics: Biochemical Perturbations in Golestan Cohort Study Opium Users Inform Intervention Strategies
Source: Front Nutr. 2020 Dec 22;7:584585. doi: 10.3389/fnut.2020.584585 (PMC7783045; doi:10.3389/fnut.2020.584585)
Supplement: Supplementary file 1 [file Data_Sheet_1.docx]

***Supplementary Material***

Untargeted Metabolomics: Biochemical Perturbations in Golestan Cohort Study Opium Users Inform Intervention Strategies

**Yuan-Yuan Li^1,#^, Reza Ghanbari^1,2,#^, Wimal Pathmasiri^1,#^, Susan McRitchie^1^, Hossein Poustchi^2^, Amaneh Shayanrad^2^, Gholamreza Roshandel^3^, Arash Etemadi^4^, Jonathan D. Pollock^5^, Reza Malekzadeh^2,*^, and Susan CJ Sumner^1,*^**

^1^Department of Nutrition, Nutrition Research Institute, University of North Carolina at Chapel Hill, Chapel Hill, NC, USA

^2^Digestive Oncology Research Center, Digestive Diseases Research Institute, Tehran University of Medical Science, Tehran, Iran

^3^Golestan Research Center of Gastroenterology and Hepatology, Golestan University of Medical Sciences, Gorgan, Iran

^4^Division of Cancer Epidemiology and Genetics, National Cancer Institute, Bethesda, MD, USA

^5^Genetics, Epigenetics, and Developmental Neuroscience Branch, National Institute on Drug Abuse (NIDA), Bethesda, MD, USA

# Co-first authors

*** Correspondence:**

Corresponding Author for Metabolomics, Statistics, Pathways, Biochemical Interpretations
Susan CJ Sumner, PhD
[susan_sumner@unc.edu](mailto:susan_sumner@unc.edu)

Corresponding Author for Epidemiology and Clinical Assessments
Reza Malekzadeh, MD, PhD
[dr.reza.malekzadeh@gmail.com](mailto:dr.reza.malekzadeh@gmail.com)

Checklist of *Supplementary Material*:

1). Sample preparation, data acquisition, data preprocessing and metabolite identification and annotation for untargeted metabolomics via UPLC high resolution mass spectrometry.

2). Sample preparation, data acquisition, data preprocessing and metabolite annotation for untargeted metabolomics via NMR Spectroscopy.

3). Supplementary Table 1. LC-MS: Signals that differentiated opium users from controls that were identified or annotated using the in-house physical standards library or annotated using public databases

4). Supplementary Table 2. NMR: Signals that differentiated opium users from controls that were annotated using Chenomx Software.

4). Supplementary Table 3. Candidate pathways, based on the similarity of m/z using Mummichog, that differentiated opium users from controls.

**1) Sample preparation, data acquisition, data preprocessing and metabolite identification and annotation for untargeted metabolomics via UPLC high resolution mass spectrometry.**

Urine samples were prepared according to the published methods (Want et al., 2010), with modification. In brief, 50-µL of urine sample was mixed with 400-µL methanol containing 500 ng/ml L-tryptophan-d5, and vortex at 5,000 rpm for 2 min. Quality control samples (QC pool) were prepared by pooling 7-µL urine from each of the study samples and processed identically to the study samples. Study samples and QC pools were centrifuged at 16, 000 rcf for 5 min at 4°C. The supernatant (320-µL) was dried and reconstituted with 100 µl water-methanol (95:5, v/v). The study samples were randomized with interspersed QC pools before data acquisition.

Metabolomics data was acquired on a Vanquish UHPLC systems coupled with a Q Exactive™ HF-X Hybrid Quadrupole-Orbitrap™ Mass Spectrometer (UPLC-HR-MS; Thermo Fisher Scientific, San Jose, CA). Metabolites were separated via an HSS T3 C18 column (2.1 x 100 mm, 1.7 µm, Waters Corporation) at 50 °C with binary mobile phases, which are water (A) and methanol (B), each containing 0.1% formic acid (v/v). The UHPLC linear gradient started from 2% B, and increased to 100% B in 16 min, then held for 4 min, with a flow rate at 0.4 ml/min. The untargeted data was collected from 70 to 1050 m/z under the data dependent acquisition (DDA) mode.

The untargeted data was processed by Progenesis QI (version 2.1, Waters Corporation) for peak picking, alignment, and normalization. The highly varied signal (peak) with RSD>30% across QC pools, and the signal with missing value in all QC pools were excluded for further analysis. Signals that highly varied (RSD>30%) or that were missing across the QC Pools were excluded for further analysis. Peaks were normalized in Progenesis QI using the “normalize to total intensity” feature.

Metabolite Identification and Annotation: Peaks detected by UPLC-HR-MS were identified or annotated through matching to an in-house experimental standards library generated by acquiring data for over 1,000 compounds under identical conditions to the study samples, as well as to public database (including HMDB, METLIN, and NIST). Identifications and annotations used available data for retention time (RT), exact mass (MS), MS/MS fragmentation pattern, and isotopic pattern. Signals/metabolites reported in the results and discussion section that matched to the in-house experimental standards library by (a) RT, MS, and MS/MS are labeled as OL1, or (b) by RT and MS are labeled OL2a. An OL2b label was provided for signals that match by MS and MS/MS to the in-house library, that were outside the retention time tolerance (±0.5 min) for the standards run under identical conditions. Signals matched to public databases are labeled as PDa (MS and experimental MS/MS), PDb (MS and theoretical MS/MS), and PDc (mass match) are also provided in supplemental material.

**2) Sample preparation, data acquisition, data preprocessing and metabolite annotation for untargeted metabolomics via NMR spectroscopy.**

Urine samples were prepared according to the published methods (Beckonert et al., 2007; Loeser et al., 2016). In brief, Aliquots of 400 µL of study samples were transferred to 2.0mL pre-labeled LoBind Eppendorf tubes. The aliquots were mixed with 300uL of NMR Master Mix solution containing Chenomx ISTD: DSS-d6 and Phosphate Buffer at 7.4 pH in D_2_O (0.5 mM final DSS-d6 concentration in NMR sample). The tubes were vortexed for 5 min on a multi-tube vortexer and centrifuged at 16,000 rcf for 10 min. A 600uL aliquot of supernatants were transferred into a pre-labeled 5mm 4" NMR tubes for data acquisition on a 700 MHz spectrometer. Six aliquots of 400 µL of analytical quality control (QC) external pooled urine samples (CHEAR Reference Pooled Urine) per batch were also used and processed identical to the study samples and used for QC purposes only.

^1^H NMR spectra of urine samples were acquired on a Bruker Avance III 700 MHz NMR spectrometer using a 5 mm cryogenically cooled ATMA inverse probe and ambient temperature of 25 ℃. A 1D NOESY pre-saturation pulse sequence (noesygppr1d, [recycle delay (RD)-90°-t_1_-90°-t_m_-90°-acquire free induction decay (FID) was used for data acquisition. For each sample 32 transients were collected into 64k data points using a spectral width of 12ppm), 2 s relaxation delay, 10 ms mixing time, and an acquisition time of 3.893 s per FID. The water resonance was suppressed using resonance irradiation during the relaxation delay and mixing time. NMR spectra were processed using TopSpin 3.5 software (Bruker-Biospin, Germany). Spectra were zero filled, and Fourier transformed after exponential multiplication with line broadening factor of 0.5 Hz. Phase and baseline of the spectra were manually corrected for each spectrum. Spectra were referenced internally to the DSS-d_6_ signal. Quality control measures included review of each NMR spectrum for line shape and width, phase and baseline of spectra, and tight clustering of QC samples in Principal Component Analysis (Broadhurst et al., 2018). NMR bins (0.50-10.00ppm) were made after excluding water (4.69 – 4.90ppm) and urea (5.50 – 6.00ppm), using intelligent bucket Integration with a 0.04 ppm bucket width and 50% looseness using ACD Spectrus 2017 Processor (ACD Labs Inc, Toronto, Canada). Integrals of each of the bins were normalized to total integral of each of the spectrum. The normalized NMR bin data were further subject to multivariate data analysis and statistical analyses (see main text). The NMR bins deemed to be important for differentiation of study phenotypes were library matched to metabolites using Chenomx NMR Suite 8.1 Professional software (Chenomx, Edmonton, Alberta, Canada) (Weljie et al., 2006).

**3).** **Supplementary Table 1. LC-MS: Signals that differentiated opium users from controls that were identified or annotated using the in-house physical standards library or annotated using public databases**

| **Metabolite/Peak**  **(3736 signals, 451 with identification or annotation in OL1, OL2a, OL2b, and PDa)**^a^ | **Ontology**^b^ | **VIP**^c^ | **FC**^d^ | ***p*-value**^e^ |
| --- | --- | --- | --- | --- |
| Morphine-6-beta-D-glucuronide | OL1 | 2.3 | 139.1 | <.0001 |
| Morphine-3-beta-D-glucuronide | OL1 | 2.3 | 117.4 | <.0001 |
| Morphine | OL1 | 2.3 | 47.0 | <.0001 |
| Codeine | OL1 | 2.5 | 42.9 | <.0001 |
| Codeine-6-beta-D-glucuronide | OL1 | 2.6 | 25.5 | <.0001 |
| Codeine | OL1 | 2.7 | 22.6 | <.0001 |
| FERULATE | OL1 | 2.1 | 5.6 | <.0001 |
| Trans-3'-Hydroxycotinine | OL1 | 1.7 | 5.3 | <.0001 |
| Caffeic acid | OL1 | 1.0 | 4.8 | <.0001 |
| Nicotine | OL1 | 1.4 | 4.5 | <.0001 |
| Cotinine | OL1 | 1.6 | 3.8 | <.0001 |
| (1'S,2'S)-Nicotine 1'-Oxide | OL1 | 1.4 | 3.7 | <.0001 |
| N-Acetyl-S-(carbamoylethyl) -L-cysteine | OL1 | 2.0 | 3.2 | <.0001 |
| N-Acetyl-S-(3-hydroxypropyl)cysteine | OL1 | 1.5 | 2.3 | <.0001 |
| Pyridoxine | OL1 | 1.9 | 1.8 | <.0001 |
| Phenethylamine | OL1 | 1.1 | 1.6 | <.0001 |
| ALLOTHREONINE | OL1 | 1.4 | 1.4 | <.0001 |
| N-ACETYLCYSTEINE | OL1 | 1.5 | 1.4 | <.0001 |
| Homovanillic acid | OL1 | 1.6 | -1.3 | <.0001 |
| L-Tryptophan | OL1 | 1.5 | -1.4 | <.0001 |
| PANTOTHENATE | OL1 | 1.9 | -1.4 | <.0001 |
| L-Tyrosine | OL1 | 1.4 | -1.4 | <.0001 |
| N-Methyl-L-glutamic acid | OL1 | 1.6 | -1.5 | <.0001 |
| 3,5 dihydroxybenzyl alcohol | OL1 | 1.4 | -1.5 | <.0001 |
| DL-Leucine | OL1 | 1.7 | -1.6 | <.0001 |
| L-Isoleucine | OL1 | 1.4 | -1.4 | <.001 |
| N-Acetyl-DL-tryptophan | OL1 | 1.6 | -1.6 | <.001 |
| N-ACETYLGLUTAMATE | OL1 | 1.4 | -1.2 | <.001 |
| 1-Methyl-L-histidine | OL1 | 0.8 | 1.3 | 0.001 |
| N-ACETYLASPARAGINE | OL1 | 1.2 | -1.3 | 0.001 |
| N-Acetyl-S-(3,4-dihydroxybutyl) -L-cysteine | OL1 | 1.3 | 1.3 | 0.001 |
| Biotin | OL1 | 1.2 | -1.5 | 0.004 |
| MEVALONATE | OL1 | 1.0 | -1.3 | 0.004 |
| p-Methylhippuric acid | OL1 | 0.7 | 1.7 | 0.006 |
| 10-hydroxydecanoic acid | OL1 | 1.1 | -1.3 | 0.006 |
| GLUCURONATE | OL1 | 0.9 | 1.3 | 0.007 |
| L-CARNITINE | OL1 | 0.9 | -1.5 | 0.008 |
| PIPECOLATE | OL1 | 1.1 | -1.8 | 0.009 |
| N-ACETYLLEUCINE | OL1 | 0.9 | -1.3 | 0.010 |
| N,N-DIMETHYL-ARGININE | OL1 | 1.1 | 1.1 | 0.012 |
| Cytidine | OL1 | 1.3 | -1.1 | 0.013 |
| Creatine | OL1 | 0.9 | -1.8 | 0.014 |
| S-ADENOSYLHOMOCYSTEINE | OL1 | 1.2 | 1.2 | 0.016 |
| 4-HYDROXY-3-METHOXYPHENYLGLYCOL | OL1 | 1.1 | -1.4 | 0.018 |
| AZELATE | OL1 | 0.9 | -1.4 | 0.020 |
| ANTHRANILATE | OL1 | 0.8 | -1.5 | 0.021 |
| Succinic acid | OL1 | 1.1 | -1.2 | 0.022 |
| O-ACETYLCARNITINE | OL1 | 0.9 | -1.7 | 0.028 |
| 4-Hydroxyhippuric acid | OL1 | 0.7 | 1.3 | 0.029 |
| N-ACETYLLEUCINE | OL1 | 0.8 | -1.1 | 0.029 |
| L-Methionine | OL1 | 1.0 | -1.2 | 0.031 |
| 2-aminophenol | OL1 | 0.7 | 1.2 | 0.032 |
| SUBERATE | OL1 | 0.9 | -1.3 | 0.033 |
| 3,4-Dihydroxybenzaldehyde | OL1 | 0.7 | 1.3 | 0.035 |
| Pyroglutamic acid | OL1 | 1.1 | -1.1 | 0.035 |
| 4-Pyridoxic acid | OL1 | 1.0 | -1.1 | 0.038 |
| RAFFINOSE | OL1 | 0.6 | -1.4 | 0.041 |
| 3-Hydroxy-3-methylglutaric acid | OL1 | 1.0 | -1.2 | 0.046 |
| CORTISOL | OL1 | 0.5 | 1.3 | 0.051 |
| XANTHURENATE | OL1 | 0.9 | -1.1 | 0.058 |
| N-Acetyl-D-galactosamine | OL1 | 1.0 | 1.1 | 0.065 |
| 10-hydroxydecanoic acid | OL1 | 0.6 | -1.3 | 0.065 |
| TRIGONELLINE | OL1 | 0.6 | -1.2 | 0.067 |
| TRYPTAMINE | OL1 | 0.8 | -1.1 | 0.073 |
| Betaine | OL1 | 0.7 | -1.3 | 0.074 |
| Adenine | OL1 | 0.8 | -1.5 | 0.076 |
| 2,6-Diaminopimelic acid | OL1 | 0.5 | 1.3 | 0.082 |
| ADENOSINE | OL1 | 1.0 | 1.1 | 0.084 |
| N-METHYLTRYPTAMINE | OL1 | 0.6 | -1.4 | 0.085 |
| N-ACETYLSERINE | OL1 | 0.7 | 1.1 | 0.094 |
| Hippuric acid | OL1 | 0.6 | -1.1 | 0.095 |
| Codeine isomer or derivative | OL2A | 2.2 | 24.9 | <.0001 |
| Monoisopropyl phthalate | OL2A | 1.1 | 16.2 | <.0001 |
| DL-2-Aminoadipic acid | OL2A | 2.0 | 3.8 | <.0001 |
| INDOLEACETALDEHYDE | OL2A | 1.8 | 3.5 | <.0001 |
| 3,4,5-trimethoxybenzaldehyde | OL2A | 0.8 | 2.7 | <.0001 |
| ITACONATE | OL2A | 1.4 | 2.3 | <.0001 |
| INDOLE-3-ETHANOL | OL2A | 2.0 | 2.2 | <.0001 |
| Taurine | OL2A | 1.2 | 1.6 | <.0001 |
| PYRIDOXAL | OL2A | 1.4 | 1.6 | <.0001 |
| 3-METHOXYTYRAMINE | OL2A | 1.8 | 1.5 | <.0001 |
| 2-ACETAMIDO-2-DEOXY-BETA-D-GLUCOSYLAMINE | OL2A | 1.5 | 1.5 | <.0001 |
| N-Acetyl-S-(3,4-dihydroxybutyl) -L-cysteine | OL2A | 1.4 | 1.4 | <.0001 |
| N-ACETYLPUTRESCINE | OL2A | 1.7 | 1.3 | <.0001 |
| 3-METHYLHISTAMINE | OL2A | 1.2 | 1.3 | <.0001 |
| Guanidineacetic acid | OL2A | 1.7 | -1.6 | <.0001 |
| 5-Methylcytosine hydrochloride | OL2A | 1.6 | -1.7 | <.0001 |
| N-ACETYLPHENYLALANINE | OL2A | 1.6 | -1.9 | <.001 |
| Mono-2-ethyl-5-hydroxyhexyl phthalate | OL2A | 1.6 | -1.9 | 0.001 |
| L-Proline | OL2A | 1.1 | 1.8 | 0.002 |
| DEOXYADENOSINE | OL2A | 1.2 | -1.2 | 0.003 |
| Homoveratric acid | OL2A | 1.1 | -1.2 | 0.003 |
| Threonine | OL2A | 0.9 | -1.5 | 0.004 |
| 3-(carbamoylamino)propanoic acid | OL2A | 1.3 | -1.3 | 0.004 |
| 6-CARBOXYHEXANOATE | OL2A | 1.0 | -1.3 | 0.004 |
| METHYGLUTARATE | OL2A | 1.1 | -1.2 | 0.004 |
| ANSERINE | OL2A | 0.9 | -2.1 | 0.005 |
| 4-acetamidobutanoic acid | OL2A | 1.3 | -1.1 | 0.005 |
| Nicotinamide | OL2A | 0.5 | 1.7 | 0.007 |
| ANILINE-2-SULFONATE | OL2A | 1.1 | -1.4 | 0.009 |
| Syringic acid | OL2A | 1.1 | -1.2 | 0.011 |
| METHYL GALACTOSIDE | OL2A | 1.2 | -2.3 | 0.014 |
| Kynurenine | OL2A | 0.9 | -1.3 | 0.014 |
| 1-METHYLADENOSINE | OL2A | 1.0 | -1.5 | 0.020 |
| N-Acetyl-S-(3,4-dihydroxybutyl) -L-cysteine | OL2A | 0.5 | 3.1 | 0.024 |
| N-ACETYLPHENYLALANINE | OL2A | 0.7 | 1.6 | 0.024 |
| 3,4,5-trimethoxybenzaldehyde | OL2A | 0.9 | -1.6 | 0.024 |
| Cytidine | OL2A | 0.7 | 1.2 | 0.029 |
| 5-HYDROXYTRYPTOPHAN | OL2A | 1.2 | -1.2 | 0.029 |
| N-ACETYLALANINE | OL2A | 1.0 | -1.1 | 0.029 |
| N-ACETYLPROLINE | OL2A | 1.0 | -1.2 | 0.031 |
| Uridine | OL2A | 0.9 | -1.2 | 0.035 |
| Creatinine | OL2A | 1.2 | -1.1 | 0.038 |
| Hydrocinnamic acid | OL2A | 0.5 | 1.9 | 0.049 |
| O-ACETYLCARNITINE | OL2A | 0.7 | 1.2 | 0.049 |
| Monoethyl phthalate | OL2A | 1.0 | -1.2 | 0.049 |
| MANNOSE | OL2A | 0.9 | -4.1 | 0.051 |
| Homoveratric acid | OL2A | 0.8 | -1.4 | 0.080 |
| ESTRADIOL-17ALPHA | OL2A | 0.5 | 1.4 | 0.081 |
| SEBACATE | OL2A | 1.0 | -1.2 | 0.090 |
| 6-Hydroxypyridine-3-carboxylic acid | OL2A | 1.0 | -1.2 | 0.094 |
| Morphine-3-beta-D-glucuronide | OL2B | 2.4 | 296.5 | <.0001 |
| Dihydromorphine | OL2B | 2.0 | 62.3 | <.0001 |
| Morphine | OL2B | 2.2 | 39.3 | <.0001 |
| Anatabine | OL2B | 1.9 | 38.4 | <.0001 |
| Morphine-3-beta-D-glucuronide | OL2B | 2.6 | 25.6 | <.0001 |
| SEROTONIN | OL2B | 1.4 | 14.0 | <.0001 |
| KYNURENATE | OL2B | 1.4 | 5.6 | <.0001 |
| 2-isopropyl-6-methyl-pyrimidinol | OL2B | 1.7 | 5.3 | <.0001 |
| SALSOLINOL | OL2B | 1.5 | 5.2 | <.0001 |
| (1'S,2'S)-Nicotine 1'-Oxide | OL2B | 1.9 | 5.0 | <.0001 |
| Trans-3'-Hydroxycotinine | OL2B | 1.6 | 2.8 | <.0001 |
| P-OCTOPAMINE | OL2B | 1.6 | 2.3 | <.0001 |
| N-ACETYLLEUCINE | OL2B | 1.2 | 2.1 | <.0001 |
| D-(+)-Glucuronic acid Î³-lactone | OL2B | 1.5 | 2.0 | <.0001 |
| N-ACETYLCYSTEINE | OL2B | 1.4 | 2.0 | <.0001 |
| N-ACETYLCYSTEINE | OL2B | 1.7 | 2.0 | <.0001 |
| Monomethyl phthalate | OL2B | 1.0 | 1.4 | <.0001 |
| N-ACETYLCYSTEINE | OL2B | 1.3 | 1.3 | <.0001 |
| PYRIDOXAL | OL2B | 1.4 | 1.3 | <.0001 |
| 12-Hydroxydodecanoic acid | OL2B | 1.1 | 1.2 | <.0001 |
| DOPAMINE | OL2B | 1.3 | 1.2 | <.0001 |
| 6-HYDROXYDOPAMINE | OL2B | 1.2 | 1.2 | <.0001 |
| Benzylamine | OL2B | 1.5 | -1.3 | <.0001 |
| CORTISOL | OL2B | 1.6 | -1.4 | <.0001 |
| Naloxone-3-beta-D-glucuronide | OL2B | 1.7 | ∞ | <.0001 |
| 2-HYDROXYBUTYRATE | OL2B | 1.5 | -1.2 | <.001 |
| 3-METHYLHISTAMINE | OL2B | 0.9 | 1.7 | <.001 |
| Î³,Î³-Dimethylallyl pyrophosphate triammonium salt | OL2B | 1.4 | -1.3 | <.001 |
| D-(+)-Glucosamine | OL2B | 0.6 | 1.3 | 0.001 |
| 2-HYDROXY-4-(METHYLTHIO)BUTANOATE | OL2B | 1.4 | -1.5 | 0.001 |
| trans-Cinnamic acid | OL2B | 1.2 | -1.2 | 0.001 |
| N-ACETYLLEUCINE | OL2B | 0.8 | 2.6 | 0.001 |
| 4-Methylcatechol | OL2B | 1.5 | -1.8 | 0.001 |
| Homovanillic acid | OL2B | 1.2 | -1.5 | 0.001 |
| P-OCTOPAMINE | OL2B | 1.3 | 1.2 | 0.002 |
| GLYCOCHENODEOXYCHOLATE | OL2B | 0.9 | -1.5 | 0.002 |
| 3,5 dihydroxybenzyl alcohol | OL2B | 0.9 | 1.2 | 0.003 |
| Glutamine | OL2B | 1.2 | 1.2 | 0.003 |
| ITACONATE | OL2B | 1.0 | 1.2 | 0.004 |
| 10-Hydroxymorphine | OL2B | 0.8 | 19.3 | 0.004 |
| trans-Cinnamic acid | OL2B | 0.8 | 1.5 | 0.004 |
| INDOLE-3-ACETAMIDE | OL2B | 1.3 | 1.2 | 0.005 |
| 3,4-Dimethoxybenzaldehyde | OL2B | 1.2 | -1.5 | 0.006 |
| URSODEOXYCHOLATE | OL2B | 1.0 | 1.2 | 0.006 |
| Adenine | OL2B | 0.9 | -1.3 | 0.006 |
| SEBACATE | OL2B | 1.1 | -1.2 | 0.007 |
| Hydrocinnamic acid | OL2B | 0.9 | 1.2 | 0.007 |
| PYRIDOXAL | OL2B | 1.1 | -1.4 | 0.007 |
| N-ACETYLLEUCINE | OL2B | 0.7 | 1.8 | 0.011 |
| (1'S,2'S)-Nicotine 1'-Oxide | OL2B | 1.2 | -3.2 | 0.011 |
| N-ACETYLPROLINE | OL2B | 0.8 | -1.2 | 0.012 |
| PYRIDOXAL | OL2B | 1.0 | -1.2 | 0.014 |
| Homoveratric acid | OL2B | 1.2 | -2.5 | 0.015 |
| 5-HYDROXYINDOLEACETATE | OL2B | 0.8 | 1.2 | 0.016 |
| HOMOGENTISATE | OL2B | 1.0 | -1.4 | 0.019 |
| MEVALONATE | OL2B | 0.8 | -1.8 | 0.020 |
| Sinapic acid | OL2B | 1.0 | -1.5 | 0.023 |
| MEVALONATE | OL2B | 0.7 | -1.8 | 0.024 |
| Adenine | OL2B | 0.8 | -1.2 | 0.026 |
| MEVALONATE | OL2B | 0.9 | -1.4 | 0.028 |
| 4-Methylcatechol | OL2B | 1.0 | 1.1 | 0.028 |
| Monomethyl phthalate | OL2B | 1.4 | 1.2 | 0.028 |
| Methyl jasmonate | OL2B | 1.0 | -1.2 | 0.031 |
| 3,4,5-trimethoxybenzaldehyde | OL2B | 1.0 | -1.2 | 0.034 |
| GLUTARATE | OL2B | 0.6 | 1.2 | 0.035 |
| 6-CARBOXYHEXANOATE | OL2B | 0.8 | 1.4 | 0.037 |
| 3-HYDROXYANTHRANILATE | OL2B | 1.1 | -1.2 | 0.046 |
| 4-Methoxycinnamic acid | OL2B | 1.0 | -4.4 | 0.049 |
| SUBERATE | OL2B | 1.1 | -1.1 | 0.050 |
| DEOXYCHOLATE | OL2B | 0.6 | -1.3 | 0.053 |
| L-Serine | OL2B | 0.8 | -1.4 | 0.054 |
| Cortisone | OL2B | 1.0 | -1.2 | 0.062 |
| 3,5 dihydroxybenzyl alcohol | OL2B | 0.7 | -1.2 | 0.065 |
| Syringic acid | OL2B | 0.5 | 1.2 | 0.068 |
| trans-Cinnamic acid | OL2B | 0.5 | 1.3 | 0.073 |
| Biotin | OL2B | 0.7 | 1.2 | 0.073 |
| GLUTARATE | OL2B | 0.6 | 1.1 | 0.085 |
| SORBATE | OL2B | 0.6 | 1.2 | 0.092 |
| L-Citrulline | OL2B | 0.8 | 1.2 | 0.094 |
| Gallic acid | OL2B | 0.4 | 1.5 | 0.094 |
| 4',7-Dimethoxy-8-methylisoflavone | PDa | 1.8 | 2892.2 | <.0001 |
| 2-Benzoylbenzoic acid | PDa | 1.7 | 394.8 | <.0001 |
| 7,8-Dihydroxycoumarin | PDa | 1.4 | 287.1 | <.0001 |
| 2'-Hydroxy-3-methoxychalcone | PDa | 1.9 | 260.2 | <.0001 |
| 20-HETE ethanolamide | PDa | 2.0 | 205.3 | <.0001 |
| 2'-Methoxy-6-methylflavone | PDa | 2.0 | 132.8 | <.0001 |
| 3-Phenylcoumarin | PDa | 1.9 | 90.6 | <.0001 |
| 3-Phenylcoumarin | PDa | 1.6 | 88.5 | <.0001 |
| Noscapine | PDa | 1.7 | 77.4 | <.0001 |
| N-lactoyl-phenylalanine | PDa | 1.7 | 24.2 | <.0001 |
| 2'-Hydroxy-3-methoxychalcone | PDa | 1.9 | 18.3 | <.0001 |
| HYDROCOTARNINE | PDa | 1.4 | 17.5 | <.0001 |
| 4-Methyl-3-phenylcoumarin | PDa | 1.7 | 16.6 | <.0001 |
| 7,8-Dihydroxyflavone | PDa | 1.3 | 14.6 | <.0001 |
| N-Acetylneuraminic Acid, 2,3-Dehydro-2-deoxy- | PDa | 2.1 | 13.6 | <.0001 |
| EXALAMIDE | PDa | 1.7 | 13.0 | <.0001 |
| 3'-Hydroxyflavone | PDa | 1.6 | 11.9 | <.0001 |
| 7,8-Dihydroxycoumarin | PDa | 1.3 | 8.2 | <.0001 |
| (R)-(-)-2-Phenylglycinol | PDa | 0.8 | 7.5 | <.0001 |
| Phe Leu Leu | PDa | 1.8 | 7.3 | <.0001 |
| 3-(3,4-Dimethoxyphenyl)-4-methylcoumarin | PDa | 1.8 | 6.1 | <.0001 |
| Asn Ala His | PDa | 1.3 | 5.0 | <.0001 |
| 2-Pyridylethanol | PDa | 1.4 | 4.8 | <.0001 |
| 1-Naphthoic acid | PDa | 1.5 | 4.8 | <.0001 |
| KAPA | PDa | 1.2 | 3.8 | <.0001 |
| Trp Asp | PDa | 1.4 | 3.3 | <.0001 |
| Sinapyl alcohol | PDa | 1.2 | 3.0 | <.0001 |
| N-Acetylcadaverine | PDa | 1.6 | 3.0 | <.0001 |
| 2-Imino-4-methylpiperidine | PDa | 1.6 | 2.9 | <.0001 |
| 2,6-Dimethyl-2,4E,6E-octatriene | PDa | 1.1 | 2.6 | <.0001 |
| 6-Hydroxypseudooxynicotine | PDa | 1.5 | 2.5 | <.0001 |
| 2-HYDROXYXANTHONE | PDa | 1.5 | 2.3 | <.0001 |
| Fraxetin | PDa | 1.2 | 2.0 | <.0001 |
| Cys-Gly | PDa | 1.5 | 2.0 | <.0001 |
| 2-methyl-1,3-Cyclohexanedione | PDa | 1.6 | 2.0 | <.0001 |
| 1-Methyluric acid | PDa | 1.5 | 1.9 | <.0001 |
| 4-hydroxy Nonenal Mercapturic Acid | PDa | 1.3 | 1.9 | <.0001 |
| Cys-Gly | PDa | 1.7 | 1.8 | <.0001 |
| 3-Hydroxypyridine | PDa | 1.7 | 1.8 | <.0001 |
| Carbaprostacyclin | PDa | 1.3 | 1.7 | <.0001 |
| 3-Hydroxypyridine | PDa | 1.6 | 1.7 | <.0001 |
| 1-METHYLXANTHINE | PDa | 1.3 | 1.7 | <.0001 |
| L-Theanine | PDa | 1.1 | 1.5 | <.0001 |
| Indolelactic acid | PDa | 1.2 | 1.5 | <.0001 |
| Asp His | PDa | 1.0 | 1.4 | <.0001 |
| 2',4'-Dihydroxyacetophenone | PDa | 1.1 | 1.4 | <.0001 |
| N-Carboxyethyl-.gamma.-aminobutyric acid | PDa | 1.4 | 1.3 | <.0001 |
| Glycylproline | PDa | 1.0 | 1.3 | <.0001 |
| N1-Acetylspermidine | PDa | 1.1 | 1.3 | <.0001 |
| 3-Methyl-.gamma.-butyrolactone | PDa | 1.4 | -1.3 | <.0001 |
| Citric acid | PDa | 1.5 | -1.3 | <.0001 |
| 3-Methylindole | PDa | 1.6 | -1.4 | <.0001 |
| Isobutyryl carnitine | PDa | 1.7 | -1.4 | <.0001 |
| 11-.beta.-Hydroxyandrosterone | PDa | 1.7 | -1.4 | <.0001 |
| trans-Dehydroandrosterone | PDa | 1.6 | -1.5 | <.0001 |
| 6.beta.-Hydroxytestosterone | PDa | 1.7 | -1.5 | <.0001 |
| .alpha.-CMBHC | PDa | 1.5 | -1.7 | <.0001 |
| His His | PDa | 1.8 | -1.7 | <.0001 |
| (-)-11-Nor-9-carboxy-.DELTA.9-THC | PDa | 2.0 | -1.8 | <.0001 |
| 5-Hydroxyferulate | PDa | 1.8 | -1.9 | <.0001 |
| 3,4,5-Trimethoxycinnamic acid | PDa | 1.7 | -1.9 | <.0001 |
| 2',3',4'-Trihydroxychalcone | PDa | 1.7 | ∞ | <.0001 |
| Lys Pro Leu | PDa | 1.1 | 1.2 | <.001 |
| Isoalantolactone | PDa | 1.6 | -1.8 | <.001 |
| N-Acetyl-L-glutamic acid | PDa | 1.7 | -1.2 | <.001 |
| (Â±)7-epi Jasmonic Acid | PDa | 1.2 | -1.3 | <.001 |
| 2,2'-(3-methylcyclohexane-1,1-diyl)diacetic acid | PDa | 1.3 | -1.6 | <.001 |
| .delta.-CEHC | PDa | 1.4 | -1.4 | <.001 |
| trans-Zeatin | PDa | 1.1 | 2.7 | <.001 |
| Cys-Gly | PDa | 1.5 | 2.4 | <.001 |
| 2-Propyl-4-pentenoic acid | PDa | 1.3 | -1.2 | <.001 |
| 11.beta.,17.alpha.,20.beta.,21-Tetrahydroxypregn-4-en-3-one | PDa | 1.2 | -1.3 | <.001 |
| Traumatic Acid | PDa | 1.3 | -1.6 | <.001 |
|  | PDa | 1.4 | -1.3 | <.001 |
| Butyryl-L-carnitine | PDa | 1.3 | -1.3 | <.001 |
| Î³-CEHC | PDa | 1.5 | -1.4 | <.001 |
| Glu Val | PDa | 1.5 | -1.2 | <.001 |
| CEPHALOTAXINE | PDa | 0.7 | 56.4 | <.001 |
| 5.alpha.-Androsterone | PDa | 1.5 | -1.5 | 0.001 |
| Val His Ile | PDa | 1.5 | -1.3 | 0.001 |
| Jasmonic acid | PDa | 1.4 | -1.2 | 0.001 |
| Asp Phe Ser | PDa | 1.2 | 1.2 | 0.001 |
| (Â±)-Hexanoylcarnitine | PDa | 1.5 | -1.4 | 0.001 |
| 5-Hydroxyferulate | PDa | 1.5 | -1.6 | 0.001 |
| N-Carboxyethyl-.gamma.-aminobutyric acid | PDa | 0.9 | 1.4 | 0.001 |
| 4-Androsten-17.beta.-ol-3-one glucosiduronate | PDa | 1.5 | -1.4 | 0.001 |
| Cadalene | PDa | 1.4 | -1.5 | 0.001 |
| Oxindole | PDa | 1.4 | -1.6 | 0.001 |
| Kavain, DL- | PDa | 1.0 | -1.3 | 0.001 |
| 4-Androsten-17.beta.-ol-3-one glucosiduronate | PDa | 1.5 | -1.5 | 0.001 |
| Glycerophosphocholine | PDa | 0.8 | 1.4 | 0.001 |
| N-Acetylneuraminic Acid, 2,3-Dehydro-2-deoxy- | PDa | 1.5 | -1.2 | 0.001 |
| 11Î±-hydroxy Testosterone | PDa | 1.4 | -1.3 | 0.001 |
| Î”17-U-46619 | PDa | 1.3 | -1.2 | 0.001 |
| 17-phenyl trinor Prostaglandin F2Î± serinol amide | PDa | 1.1 | -2.3 | 0.001 |
| N2-Acetyl-L-ornithine | PDa | 1.3 | 1.5 | 0.001 |
| 5-Hydroxyferulate | PDa | 0.8 | 16.8 | 0.001 |
| 6-tert-Butyl-2H-1,2,4-triazine-3,5-dione | PDa | 1.3 | -1.6 | 0.002 |
| N-Methyltyramine | PDa | 1.1 | -2.6 | 0.002 |
| 4-Vinylguaiacol | PDa | 1.4 | -3.3 | 0.002 |
| Î´-Valerolactam | PDa | 1.1 | 1.5 | 0.002 |
| L-Homocitrulline | PDa | 1.2 | -1.5 | 0.002 |
| Gamma-Glu-Leu | PDa | 1.2 | -1.1 | 0.002 |
| Dihydronepetalactone | PDa | 1.0 | -1.3 | 0.002 |
| Cadalene | PDa | 1.2 | -1.3 | 0.002 |
| L-Tyrosinamide | PDa | 1.3 | 1.3 | 0.003 |
| Methyl 3-(4-methoxyphenyl)-2-oxopropanoate | PDa | 0.6 | 1.8 | 0.003 |
| L-Allysine Ethylene Acetal | PDa | 1.3 | -1.2 | 0.003 |
| 4-tert-Butylbenzylamine | PDa | 1.2 | -1.2 | 0.003 |
| 5.alpha.-Androsterone | PDa | 1.3 | -1.4 | 0.003 |
| ent-Corey PG-Lactone diol | PDa | 1.2 | -1.7 | 0.003 |
| Chamazulene | PDa | 1.3 | -1.5 | 0.004 |
| Atraric acid | PDa | 1.4 | -4.0 | 0.004 |
| Methyl jasmonate | PDa | 1.0 | -1.2 | 0.004 |
| 5-Pregnen-3.beta.-ol-20-one | PDa | 0.8 | 1.5 | 0.004 |
| 2,6-Dimethylnaphthalene | PDa | 1.2 | -1.3 | 0.004 |
| Dinor-12-oxophytodienoic Acid | PDa | 1.3 | -1.3 | 0.004 |
| N-Acetylcadaverine | PDa | 0.9 | 1.8 | 0.005 |
| Uric acid, 1,3-dimethyl- | PDa | 0.5 | 2.2 | 0.005 |
| Asp His Phe | PDa | 0.6 | 1.7 | 0.005 |
| N-Desmethyldiazepam (Nordazepam) | PDa | 0.7 | 4.5 | 0.005 |
| 4-Pregnen-11,17,20,21-tetrol-3-one | PDa | 1.3 | -1.3 | 0.005 |
| 5Î±-Androst-16-en-3Î±-ol | PDa | 1.2 | -1.5 | 0.005 |
| Myristoleic acid | PDa | 0.6 | 1.3 | 0.005 |
| Phe Thr | PDa | 1.1 | -1.4 | 0.005 |
| 2-Amino-4-methylphenol | PDa | 0.5 | 4.8 | 0.005 |
| Dodecanedioic acid | PDa | 0.9 | -2.1 | 0.005 |
| Dimethyl sulfone | PDa | 1.2 | -1.3 | 0.006 |
| Salicylic acid .beta.-D-O-glucuronide | PDa | 0.7 | 4.7 | 0.006 |
| cis-Guggulsterone | PDa | 1.2 | -1.2 | 0.006 |
| 11.beta.-Hydroxyprogesterone | PDa | 1.2 | -1.2 | 0.006 |
| 4-Methylumbelliferyl .beta.-D-glucuronide | PDa | 0.6 | 1.6 | 0.007 |
| 11-.beta.-Hydroxyandrosterone | PDa | 1.3 | -1.4 | 0.007 |
| Ile-Leu | PDa | 0.8 | 1.7 | 0.008 |
| KAPA | PDa | 0.6 | 1.7 | 0.008 |
| trans-4,5-Epoxy-2(E)-decenal | PDa | 1.0 | -1.2 | 0.008 |
| .gamma.-Hexalactone | PDa | 0.9 | -1.1 | 0.009 |
| Asp Leu Gly | PDa | 1.0 | -1.3 | 0.009 |
| Ala Gln His | PDa | 1.2 | -5.1 | 0.010 |
| Metanephrine | PDa | 1.0 | 1.2 | 0.010 |
| Isopropyl 4-hydroxybenzoate | PDa | 1.0 | -2.4 | 0.010 |
| Nicotinuric acid | PDa | 1.3 | -1.2 | 0.010 |
| 2-Amino-4-methylphenol | PDa | 1.0 | -1.4 | 0.010 |
| PyroGlu-Phe | PDa | 1.0 | 1.2 | 0.011 |
| 5Î±-Androst-16-en-3Î±-ol | PDa | 0.9 | -1.9 | 0.012 |
| alantolactone | PDa | 1.2 | -1.3 | 0.012 |
| N-Acetyl-D-lactosamine | PDa | 1.0 | 1.2 | 0.012 |
| Megastigmatrienone | PDa | 0.9 | -1.2 | 0.012 |
| 3-Phenoxy-1-propanol | PDa | 0.9 | -1.1 | 0.013 |
| Cynaratriol | PDa | 0.9 | -1.6 | 0.013 |
| 5.alpha.-Androsterone | PDa | 1.1 | -1.2 | 0.013 |
| Î´-CEHC | PDa | 1.1 | -1.2 | 0.013 |
| 5.alpha.-Androsterone | PDa | 1.2 | -1.3 | 0.013 |
| N-Phenylacetylphenylalanine | PDa | 1.0 | 1.3 | 0.013 |
| Docosahexaenoic Acid methyl ester | PDa | 0.9 | 1.5 | 0.015 |
| 2',4'-Dimethoxy-3-hydroxy-6-methylflavone | PDa | 0.5 | 1.2 | 0.015 |
| 4-Androsten-17.beta.-ol-3-one glucosiduronate | PDa | 1.2 | -1.3 | 0.015 |
| His Val | PDa | 1.1 | -1.3 | 0.016 |
| Caryophyllene epoxide | PDa | 1.2 | -1.3 | 0.016 |
| Asp Ile Ile | PDa | 0.9 | -1.1 | 0.020 |
| Asp Gly | PDa | 0.8 | 1.1 | 0.020 |
| 5alpha-pregnane-3,20-dione | PDa | 0.9 | -2.0 | 0.020 |
| CYTISINE | PDa | 0.5 | 1.7 | 0.020 |
| Neocnidilide | PDa | 1.0 | -1.2 | 0.020 |
| Ile-Glu | PDa | 0.9 | -1.3 | 0.020 |
| 3-Phenoxypropionic acid | PDa | 0.7 | 1.2 | 0.020 |
| 6-Methoxy-4-methylcoumarin | PDa | 1.1 | -1.8 | 0.021 |
| 9,10-Dihydroxy-12Z-octadecenoic acid | PDa | 0.7 | -1.5 | 0.022 |
| Quadrone | PDa | 1.0 | -2.2 | 0.022 |
| (-)-trans-C75 | PDa | 1.1 | -1.2 | 0.022 |
| Cyclopentolate | PDa | 0.9 | -2.2 | 0.024 |
| 3-Pyridinemethanol | PDa | 1.2 | -1.2 | 0.025 |
| Salicylic acid .beta.-D-O-glucuronide | PDa | 0.6 | 1.4 | 0.025 |
| trans,cis-3,6-Nonadien-1-ol | PDa | 1.1 | -1.2 | 0.026 |
| His-His | PDa | 0.8 | -1.3 | 0.027 |
| Salicylic acid .beta.-D-O-glucuronide | PDa | 0.5 | 5.0 | 0.027 |
| 7-keto DHEA | PDa | 1.0 | -1.4 | 0.028 |
| Biopterin | PDa | 1.1 | 1.1 | 0.029 |
| Benzhydrol | PDa | 0.8 | -1.4 | 0.029 |
| Pinolenic acid | PDa | 0.7 | -1.4 | 0.030 |
| 1-ACETYLPIPERIDINE | PDa | 0.8 | -1.1 | 0.031 |
| 9-Oxo-10E,12Z-octadecadienoic acid | PDa | 0.8 | -1.5 | 0.032 |
| 5-iPF2.alpha.-VI | PDa | 0.9 | -1.2 | 0.033 |
| Atenolol | PDa | 0.4 | 13.2 | 0.033 |
| Tetrahydro-L-biopterin | PDa | 0.9 | -1.2 | 0.033 |
| p-Acetaminobenzoic acid | PDa | 0.9 | 1.4 | 0.034 |
| 15-deoxy-Î´-12,14-PGJ2 | PDa | 1.2 | -1.2 | 0.034 |
| 9,10-Dihydroxy-12Z-octadecenoic acid | PDa | 0.7 | -1.5 | 0.034 |
| Serylglutamine | PDa | 0.7 | 1.2 | 0.034 |
| 4-Nitrobenzylamine | PDa | 1.1 | -1.2 | 0.035 |
| Gly Met Asp | PDa | 0.9 | -1.6 | 0.035 |
| 1-Anilino-9,10-dioxo-2-anthroic acid | PDa | 0.9 | 1.2 | 0.037 |
| His-Ala-Lys | PDa | 0.8 | -1.7 | 0.038 |
| Lys Thr Ser | PDa | 0.9 | -1.4 | 0.039 |
| His-Thr-Lys | PDa | 0.9 | -1.3 | 0.039 |
| Tetradecanedioic acid | PDa | 0.8 | -1.3 | 0.041 |
| Cimetidine | PDa | 0.5 | 334.5 | 0.041 |
| Asp-Phe | PDa | 0.8 | 1.1 | 0.043 |
| Dodecanedioic acid | PDa | 0.8 | -1.2 | 0.044 |
| Dihydroactinidiolide | PDa | 0.9 | -1.1 | 0.044 |
| Bolasterone | PDa | 1.1 | -1.3 | 0.045 |
| Dinor-12-oxophytodienoic Acid | PDa | 1.1 | -1.2 | 0.046 |
| 2-[(4-Aminobenzoyl)amino]acetic acid | PDa | 1.2 | -1.1 | 0.046 |
| Asp Ile Val | PDa | 0.8 | -1.2 | 0.047 |
| Decanoyl-L-carnitine | PDa | 1.0 | -1.8 | 0.049 |
| Î´-Valerolactam | PDa | 1.0 | -1.4 | 0.049 |
| (-)-Carveol | PDa | 0.7 | -1.6 | 0.050 |
| Isoalantolactone | PDa | 0.9 | -1.3 | 0.051 |
| 1,2-Dihexanoyl-sn-glycerol | PDa | 0.8 | -1.5 | 0.053 |
| L-Pipecolic acid | PDa | 0.7 | 1.2 | 0.054 |
| L-Tyrosinamide | PDa | 0.9 | 1.5 | 0.056 |
| Cantharidin | PDa | 0.7 | -1.5 | 0.056 |
| Myristic acid alkyne | PDa | 1.0 | 1.2 | 0.058 |
| O-Acetylsalicylhydroxamic Acid | PDa | 0.6 | 2.5 | 0.058 |
| Gln Ile | PDa | 0.8 | -3.7 | 0.058 |
| Terbutaline | PDa | 0.8 | -2.8 | 0.060 |
| 5-Androsten-3.beta.-ol-17-one | PDa | 1.1 | -1.4 | 0.062 |
| 5Î±-Androst-16-en-3Î±-ol | PDa | 0.9 | -1.2 | 0.063 |
| D-(+)-Neopterin | PDa | 0.8 | 1.2 | 0.064 |
| 1,7-Dimethyluric acid | PDa | 0.7 | 1.2 | 0.066 |
| Oxazepam | PDa | 0.5 | 2.4 | 0.067 |
| CYTISINE | PDa | 0.6 | 1.2 | 0.067 |
| (R)-4-((3S,5R,8R,9S,10S,13R,14S,17R)-3-hydroxy-4,4,10,13,14-pentamethyl-7,11-dioxohexadecahydro-1H-cyclopenta[a]phenanthren-17-yl)pentanoic acid | PDa | 0.7 | -1.3 | 0.068 |
| 5.alpha.-Androsterone | PDa | 0.9 | -1.2 | 0.069 |
| His-Thr | PDa | 0.7 | -1.3 | 0.074 |
| trans,trans-Muconic acid | PDa | 0.5 | 1.5 | 0.075 |
| Albuterol | PDa | 0.3 | 41.7 | 0.076 |
| Confertifoline | PDa | 1.0 | -1.1 | 0.078 |
| 5.alpha.-Pregnan-3.alpha.-ol-11,20-dione | PDa | 0.9 | 1.2 | 0.081 |
| Caryophyllene epoxide | PDa | 0.8 | -2.4 | 0.081 |
| 11-.beta.-Hydroxyandrosterone | PDa | 1.0 | -1.2 | 0.082 |
| 5.alpha.-Pregnan-3.alpha.-ol-11,20-dione | PDa | 0.9 | 1.2 | 0.084 |
| Asn Leu | PDa | 1.0 | 1.1 | 0.085 |
| Tibolone | PDa | 1.0 | -1.2 | 0.087 |
| Methyl jasmonate | PDa | 0.8 | -1.4 | 0.089 |
| N-formylanthranilic acid | PDa | 0.7 | -1.8 | 0.090 |
| Methyl geranate | PDa | 0.7 | -1.3 | 0.091 |
| 9S-Hydroxy-10E,12Z,15Z-octadecatrienoic acid | PDa | 0.7 | -1.9 | 0.091 |
| Acetyl-DL-Valine | PDa | 0.5 | 1.2 | 0.091 |
| Sinapyl alcohol | PDa | 0.8 | 1.2 | 0.092 |
| THYMOQUINONE | PDa | 0.7 | -1.2 | 0.093 |
| 9S-Hydroxy-10E,12Z,15Z-octadecatrienoic acid | PDa | 0.7 | -1.6 | 0.094 |
| 19(R)-HETE | PDa | 0.8 | 1.2 | 0.094 |
| Ile Gln Asp | PDa | 0.9 | 1.2 | 0.094 |
| Ala-Val-OH | PDa | 1.1 | 1.1 | 0.095 |
| Glu Leu | PDa | 1.1 | -1.2 | 0.098 |
| Pro Phe Arg | PDa | 1.1 | -1.1 | 0.099 |
| Gly Gln Ser | PDa | 0.9 | -1.2 | 0.099 |
| Biopterin | PDa | 1.0 | -1.3 | 0.100 |
| 5-Pregnen-3.beta.-ol-20-one | PDa | 0.7 | -1.2 | 0.100 |

^a^Cut-off criterion for peak/metabolite that differentiates opium users versus none users is p<0.1 (t-test). ^b^Ontology levels: OL1, highly confident identification based on matching with In-house physical standard library (IPSL) via retention time (RT, with RT error≤|0.5|), exact mass (MS, with mass error<5ppm), and tandem mass similarity (MS/MS, with similarity ≥30); OL2a, confident identification based on matching with IPSL via MS and RT; OL2b, annotation for the isomer or derivatives of the compound listed but not the compound itself, based on matching with IPSL via MS and MS/MS; PDa, annotation based on matching with public database via MS and experimental MS/MS (could be the listed compound, or the isomer or derivatives of the listed compound); ^c^VIP, variable influence on projections to latent structures; ^d^FC, fold change, the ratio of intensity between opium users versus none users based on the mean, indicates the direction and magnitude of change: FC>1.0 indicates increase level in the users compared to none user. ∞, the metabolite is not detected in the none users; ^e^p-value, determined by t-test

**Supplementary Table 2. NMR: Signals that differentiated opium users from controls that were annotated using Chenomx Software.**

| Chemical Shift | Library Matched Metabolites | Higher (+)/  Lower (-)* | VIP | p-value* | Fold Change** |
| --- | --- | --- | --- | --- | --- |
| [0.52 .. 0.57] | Unknown | - | 0.09 | 0.1994 | -1.56 |
| [0.69 .. 0.71] | Cholate \| Glycocholate | - | 0.07 | 0.2226 | -1.08 |
| [0.75 .. 0.77] | Unknown | - | 0.13 | 0.2846 | -1.05 |
| [0.81 .. 0.83] | Fatty acids | - | 0.22 | 0.435 | -1.04 |
| [0.83 .. 0.85] | Fatty acids | - | 0.26 | 0.3014 | -1.05 |
| [0.91 .. 0.97] | Isoleucine \| Leucine | - | 0.57 | 0.5078 | -1.02 |
| [0.97 .. 1.00] | Valine \| Isoleucine | - | 0.41 | 0.0376 | -1.07 |
| [1.00 .. 1.02] | Isoleucine | + | 0.19 | 0.9413 | 1.00 |
| [1.02 .. 1.08] | Valine \| Methylsuccinate | - | 0.51 | 0.0286 | -1.12 |
| [1.08 .. 1.12] | 3-Methyl-2-oxo-valerate \| Isobutyrate | + | 0.39 | 0.9041 | 1.00 |
| [1.15 .. 1.17] | Ethanol | - | 0.23 | 0.8462 | -1.02 |
| [1.21 .. 1.25] | Unknown | - | 0.74 | 0.1882 | -1.06 |
| [1.25 .. 1.31] | 3-Hydroxyisovalerate | - | 0.91 | 0.0137 | -1.13 |
| [1.31 .. 1.34] | Lactate \| Threonine | - | 0.94 | 0.0022 | -1.14 |
| [1.43 .. 1.46] | Fatty acids | - | 0.52 | 0.0018 | -1.14 |
| [1.46 .. 1.51] | Alanine | - | 0.97 | 0.0018 | -1.14 |
| [1.51 .. 1.57] | Fatty acids | - | 0.54 | 0.176 | -1.08 |
| [1.67 .. 1.69] | Fatty acids | - | 0.39 | 0.87 | -1.01 |
| [1.90 .. 1.95] | Acetate \| Lysine | + | 1.01 | 0.0061 | 1.10 |
| [1.95 .. 1.99] | N6-Acetyllysine | + | 0.61 | 0.02 | 1.08 |
| [1.99 .. 2.01] | N-Acetyl amino acids | + | 0.44 | 0.0421 | 1.07 |
| [2.01 .. 2.07] | N-Acetyl amino acids | + | 1.10 | 0.2602 | 1.04 |
| [2.15 .. 2.20] | Unnknown | + | 1.43 | 0.2793 | 1.09 |
| [2.23 .. 2.29] | Unknown | + | 1.93 | <.0001 | 1.21 |
| [2.29 .. 2.34] | Unknown \| Acetylsalicylate | + | 1.80 | <.0001 | 1.25 |
| [2.51 .. 2.56] | Citrate | - | 1.93 | 0.0028 | -1.19 |
| [2.59 .. 2.63] | Methylamine \| Methylsuccinate \| Ribiflavine | + | 0.39 | 0.049 | 1.08 |
| [2.65 .. 2.70] | Citrate | - | 1.60 | 0.0142 | -1.15 |
| [2.70 .. 2.76] | Dimethylamine | + | 0.99 | 0.0126 | 1.09 |
| [2.76 .. 2.80] | Unknown | + | 0.61 | 0.0023 | 1.15 |
| [2.90 .. 2.93] | N, N-Dimethylglycine | + | 0.58 | 0.0038 | 1.25 |
| [2.93 .. 2.95] | N-Methylhydantoin | + | 1.36 | <.0001 | 1.49 |
| [3.00 .. 3.06] | Creatinine | - | 3.82 | 0.0055 | -1.11 |
| [3.09 .. 3.12] | Malonate \| cis-Aconitate | - | 1.13 | 0.0007 | -1.24 |
| [3.16 .. 3.20] | Choline | - | 1.16 | 0.0304 | -1.12 |
| [3.20 .. 3.22] | O-Phosphocholine | - | 1.11 | 0.0021 | -1.22 |
| [3.22 .. 3.28] | Glucose \| Taurine \| Betaine \| myo-Inositol | - | 2.51 | 0.0803 | -1.12 |
| [3.37 .. 3.39] | Glucose | - | 1.20 | 0.1074 | -1.31 |
| [3.39 .. 3.43] | Glucose \| Taurine | - | 3.28 | 0.1597 | -1.35 |
| [3.43 .. 3.48] | Glucose \| p-Hydroxyphenylacetic acid | - | 3.13 | 0.1081 | -1.42 |
| [3.48 .. 3.51] | Glucose | - | 2.27 | 0.1062 | -1.29 |
| [3.51 .. 3.55] | Glucose | - | 2.07 | 0.0566 | -1.22 |
| [3.55 .. 3.57] | Glycine | - | 0.58 | 0.853 | -1.01 |
| [3.57 .. 3.60] | myo-Inositol | + | 1.46 | <.0001 | 1.19 |
| [3.60 .. 3.63] | myo-Inositol | + | 2.68 | <.0001 | 1.31 |
| [3.63 .. 3.66] | Ethanol | + | 1.56 | 0.0042 | 1.10 |
| [3.70 .. 3.74] | Glucose \| Overlapped | - | 3.40 | 0.0406 | -1.29 |
| [3.74 .. 3.80] | Glucose | - | 1.23 | 0.1233 | -1.04 |
| [3.80 .. 3.86] | Glucose | - | 2.34 | 0.896 | -1.01 |
| [3.86 .. 3.91] | Glucose | - | 2.15 | 0.804 | -1.02 |
| [3.91 .. 3.97] | Hippurate \| Glycolate | - | 1.57 | 0.4695 | -1.03 |
| [4.02 .. 4.08] | Creatinine \| myo-Inositol | - | 2.97 | 0.0086 | -1.11 |
| [4.14 .. 4.20] | O-Phosphocholine | + | 2.24 | <.0001 | 1.23 |
| [4.30 .. 4.36] | Tartrate | + | 0.61 | 0.0037 | 1.18 |
| [4.48 .. 4.54] | Unknown | + | 0.46 | 0.0447 | 1.08 |
| [4.57 .. 4.61] | Unknown | + | 0.95 | <.0001 | 1.53 |
| [4.61 .. 4.67] | Glucose | - | 2.12 | 0.1796 | -1.49 |
| [4.67 .. 4.69] | Unknown | + | 0.49 | 0.0009 | 1.49 |
| [4.94 .. 4.99] | Unknown | + | 0.15 | 0.023 | 1.17 |
| [5.08 .. 5.14] | Unknown | + | 1.88 | <.0001 | 2.52 |
| [5.18 .. 5.21] | Fucose \| N-Acetylglucosamine | - | 0.11 | 0.9402 | -1.00 |
| [5.21 .. 5.26] | Glucose | - | 2.15 | 0.0878 | -1.80 |
| [5.26 .. 5.29] | Unknown | + | 1.01 | <.0001 | 1.67 |
| [5.29 .. 5.31] | Unknown | + | 0.25 | 0.0015 | 1.17 |
| [5.36 .. 5.39] | Unknown | + | 0.66 | <.0001 | 1.30 |
| [5.39 .. 5.43] | Sucrose | + | 1.80 | <.0001 | 2.20 |
| [5.43 .. 5.46] | Unknown \| 1,6-Anhydro-beta-D-glucose | + | 0.54 | <.0001 | 1.37 |
| [5.46 .. 5.48] | Unknown \| 1,6-Anhydro-beta-D-glucose | - | 0.13 | 0.0301 | -1.13 |
| [6.00 .. 6.06] | Malate \| Unknown | + | 0.56 | 0.0002 | 1.16 |
| [6.56 .. 6.62] | trans-Aconitate | + | 0.41 | <.0001 | 1.27 |
| [6.84 .. 6.88] | p-Hydroxyphenylacetic acid | + | 1.07 | <.0001 | 1.32 |
| [6.88 .. 6.90] | Tyrosine | + | 0.34 | 0.2346 | 1.06 |
| [7.11 .. 7.17] | p-Hydroxyphenylacetic acid \| acetylsalicylate | + | 1.13 | 0.0103 | 1.31 |
| [7.17 .. 7.22] | Tyrosine | + | 1.52 | <.0001 | 1.30 |
| [7.29 .. 7.32] | Phenylalanine | + | 0.69 | 0.0643 | 1.17 |
| [7.32 .. 7.37] | N-Phenylacetylglycine \| Phenylalanine | + | 2.52 | <.0001 | 1.39 |
| [7.37 .. 7.43] | N-Phenylacetylglycine \| Phenylalanine | + | 1.87 | <.0001 | 1.35 |
| [7.48 .. 7.51] | acetylsalicylate | + | 0.47 | 0.1002 | 1.17 |
| [7.51 .. 7.57] | Hippurate | + | 0.95 | 0.7137 | 1.03 |
| [7.80 .. 7.85] | Hippurate | + | 1.04 | 0.9358 | 1.01 |
| [7.88 .. 7.93] | p-Methylhistidine \| Histidine | - | 0.19 | 0.4013 | -1.11 |
| [8.15 .. 8.21] | Hypoxanthine | + | 0.22 | 0.0543 | 1.12 |
| [8.21 .. 8.24] | Hypoxanthine \| Inosine | + | 0.31 | <.0001 | 1.56 |
| [8.31 .. 8.36] | Inosine | + | 0.22 | <.0001 | 1.32 |
| [8.80 .. 8.86] | Trigoneline | - | 0.18 | 0.0708 | -1.23 |
| [9.08 .. 9.14] | Trigonelline | + | 0.11 | 0.3053 | 1.12 |
| [9.14 .. 9.20] | Trigoneline | + | 0.02 | 0.2843 | 1.62 |

VIP=Variable Influence on Projection, All VIP values based on Pareto Scaling

*t-test (assuming unequal variances, Satterthwaite)

**All Fold Changes based on the mean; Positive denotes higher in Opium User compared to Non-user

**Supplementary Table 3. Candidate pathways, based on the similarity of m/z using Mummichog, that differentiated opium users from controls.**

| ^a^**Pathway Name** | ^b^**Pathway total** | ^c^**Hits.total** | ^d^**Hits.sig** | ^e^**Gamma** | ^f^**Pathway Number** |
| --- | --- | --- | --- | --- | --- |
| Androgen and estrogen biosynthesis and metabolism | 95 | 69 | 66 | 0.0028243 | P0 |
| Drug metabolism - cytochrome P450 | 53 | 51 | 48 | 0.0029891 | P1 |
| C21-steroid hormone biosynthesis and metabolism | 112 | 77 | 70 | 0.0030253 | P2 |
| Pentose phosphate pathway | 37 | 34 | 33 | 0.0030625 | P3 |
| Tryptophan metabolism | 94 | 69 | 63 | 0.0030679 | P4 |
| Tyrosine metabolism | 160 | 88 | 77 | 0.0038652 | P5 |
| Fructose and mannose metabolism | 33 | 25 | 23 | 0.0066839 | P6 |
| Pentose and Glucuronate Interconversions | 15 | 12 | 12 | 0.0076549 | P7 |
| Caffeine metabolism | 11 | 11 | 11 | 0.0091145 | P8 |
| Lysine metabolism | 52 | 30 | 26 | 0.014088 | P9 |
| Galactose metabolism | 41 | 35 | 30 | 0.014488 | P10 |
| Leukotriene metabolism | 92 | 47 | 39 | 0.021697 | P11 |
| Phosphatidylinositol phosphate metabolism | 59 | 22 | 19 | 0.022045 | P12 |
| Alkaloid biosynthesis II | 10 | 7 | 7 | 0.024423 | P13 |
| N-Glycan Degradation | 16 | 7 | 7 | 0.024423 | P14 |
| Urea cycle/amino group metabolism | 85 | 50 | 41 | 0.027316 | P15 |
| Sialic acid metabolism | 107 | 30 | 25 | 0.029223 | P16 |
| Hexose phosphorylation | 20 | 20 | 17 | 0.031575 | P17 |
| Starch and Sucrose Metabolism | 33 | 15 | 13 | 0.033819 | P18 |
| Heparan sulfate degradation | 34 | 6 | 6 | 0.033992 | P19 |
| Limonene and pinene degradation | 10 | 6 | 6 | 0.033992 | P20 |
| Xenobiotics metabolism | 110 | 80 | 64 | 0.041548 | P21 |
| Biopterin metabolism | 22 | 14 | 12 | 0.042139 | P22 |
| Chondroitin sulfate degradation | 37 | 5 | 5 | 0.049139 | P23 |
| Vitamin B12 (cyanocobalamin) metabolism | 9 | 5 | 5 | 0.049139 | P24 |
| Omega-3 fatty acid metabolism | 39 | 9 | 8 | 0.050039 | P25 |
| Vitamin E metabolism | 54 | 25 | 20 | 0.063798 | P26 |
| Ubiquinone Biosynthesis | 10 | 8 | 7 | 0.066703 | P27 |
| Vitamin B9 (folate) metabolism | 33 | 20 | 16 | 0.071454 | P28 |
| Vitamin H (biotin) metabolism | 5 | 4 | 4 | 0.073979 | P29 |
| Lipoate metabolism | 8 | 4 | 4 | 0.073979 | P30 |
| 1- and 2-Methylnaphthalene degradation | 4 | 4 | 4 | 0.073979 | P31 |
| Nitrogen metabolism | 6 | 4 | 4 | 0.073979 | P32 |
| Purine metabolism | 80 | 55 | 43 | 0.075612 | P33 |
| Vitamin A (retinol) metabolism | 67 | 32 | 25 | 0.081922 | P34 |
| C5-Branched dibasic acid metabolism | 10 | 7 | 6 | 0.089883 | P35 |
| Omega-6 fatty acid metabolism | 55 | 7 | 6 | 0.089883 | P36 |
| Valine, leucine and isoleucine degradation | 65 | 35 | 27 | 0.096865 | P37 |
| Prostaglandin formation from arachidonate | 78 | 61 | 47 | 0.10084 | P38 |
| Drug metabolism - other enzymes | 31 | 26 | 20 | 0.10384 | P39 |
| Glycolysis and Gluconeogenesis | 49 | 30 | 23 | 0.10631 | P40 |
| Glycosylphosphatidylinositol(GPI)-anchor biosynthesis | 6 | 3 | 3 | 0.11672 | P41 |
| Benzoate degradation via CoA ligation | 4 | 3 | 3 | 0.11672 | P42 |
| Vitamin B3 (nicotinate and nicotinamide) metabolism | 28 | 21 | 16 | 0.11876 | P43 |
| Keratan sulfate degradation | 68 | 6 | 5 | 0.12222 | P44 |
| CoA Catabolism | 7 | 6 | 5 | 0.12222 | P45 |
| N-Glycan biosynthesis | 48 | 13 | 10 | 0.12434 | P46 |
| Arginine and Proline Metabolism | 45 | 37 | 28 | 0.12537 | P47 |
| Carbon fixation | 10 | 9 | 7 | 0.13552 | P48 |
| Aminosugars metabolism | 69 | 32 | 24 | 0.13769 | P49 |
| Linoleate metabolism | 46 | 20 | 15 | 0.13783 | P50 |
| Ascorbate (Vitamin C) and Aldarate Metabolism | 29 | 20 | 15 | 0.13783 | P51 |
| Glycerophospholipid metabolism | 156 | 44 | 33 | 0.14449 | P52 |
| Squalene and cholesterol biosynthesis | 55 | 19 | 14 | 0.15939 | P53 |
| Prostaglandin formation from dihomo gama-linoleic acid | 11 | 5 | 4 | 0.16761 | P54 |
| Putative anti-Inflammatory metabolites formation from EPA | 27 | 22 | 16 | 0.17681 | P55 |
| Hyaluronan Metabolism | 8 | 2 | 2 | 0.1978 | P56 |
| Geraniol degradation | 2 | 2 | 2 | 0.1978 | P57 |
| Trihydroxycoprostanoyl-CoA beta-oxidation | 9 | 2 | 2 | 0.1978 | P58 |
| Aspartate and asparagine metabolism | 114 | 69 | 51 | 0.20447 | P59 |
| Histidine metabolism | 33 | 24 | 17 | 0.21665 | P60 |
| Vitamin B6 (pyridoxine) metabolism | 11 | 7 | 5 | 0.2168 | P61 |
| Nucleotide Sugar Metabolism | 7 | 4 | 3 | 0.23255 | P62 |
| Vitamin B2 (riboflavin) metabolism | 8 | 6 | 4 | 0.27384 | P63 |
| Fatty acid oxidation, peroxisome | 28 | 6 | 4 | 0.27384 | P64 |
| Vitamin B5 - CoA biosynthesis from pantothenate | 12 | 11 | 7 | 0.30758 | P65 |
| Di-unsaturated fatty acid beta-oxidation | 26 | 8 | 5 | 0.31498 | P66 |
| De novo fatty acid biosynthesis | 106 | 26 | 17 | 0.34558 | P67 |
| Glycosphingolipid biosynthesis - globoseries | 16 | 5 | 3 | 0.34712 | P68 |
| Parathio degradation | 6 | 5 | 3 | 0.34712 | P69 |
| Beta-Alanine metabolism | 20 | 13 | 8 | 0.34892 | P70 |
| Glycosphingolipid biosynthesis - ganglioseries | 62 | 10 | 6 | 0.35452 | P71 |
| Vitamin B1 (thiamin) metabolism | 20 | 10 | 6 | 0.35452 | P72 |
| Glutamate metabolism | 15 | 12 | 7 | 0.39205 | P73 |
| Pyrimidine metabolism | 70 | 45 | 30 | 0.39738 | P74 |
| Polyunsaturated fatty acid biosynthesis | 21 | 9 | 5 | 0.40743 | P75 |
| Methionine and cysteine metabolism | 94 | 48 | 32 | 0.40997 | P76 |
| Porphyrin metabolism | 43 | 23 | 14 | 0.4292 | P77 |
| R Group Synthesis | 7 | 4 | 2 | 0.44768 | P78 |
| Keratan sulfate biosynthesis | 66 | 4 | 2 | 0.44768 | P79 |
| Fatty Acid Metabolism | 63 | 19 | 11 | 0.45807 | P80 |
| D4&E4-neuroprostanes formation | 37 | 19 | 11 | 0.45807 | P81 |
| Alanine and Aspartate Metabolism | 30 | 22 | 13 | 0.45912 | P82 |
| Glutathione Metabolism | 19 | 13 | 7 | 0.47006 | P83 |
| Mono-unsaturated fatty acid beta-oxidation | 19 | 10 | 5 | 0.49089 | P84 |
| Phytanic acid peroxisomal oxidation | 34 | 12 | 6 | 0.51567 | P85 |
| TCA cycle | 31 | 20 | 11 | 0.52192 | P86 |
| Glycine, serine, alanine and threonine metabolism | 88 | 52 | 33 | 0.53224 | P87 |
| Fatty acid oxidation | 35 | 7 | 3 | 0.53761 | P88 |
| Butanoate metabolism | 34 | 27 | 15 | 0.57475 | P89 |
| Pyruvate Metabolism | 20 | 13 | 6 | 0.58366 | P90 |
| Propanoate metabolism | 31 | 18 | 9 | 0.58857 | P91 |
| Glyoxylate and Dicarboxylate Metabolism | 12 | 6 | 2 | 0.62385 | P92 |
| Fatty acid activation | 74 | 22 | 11 | 0.63282 | P93 |
| Selenoamino acid metabolism | 35 | 14 | 6 | 0.64347 | P94 |
| Glycosphingolipid metabolism | 67 | 28 | 14 | 0.6915 | P95 |
| Saturated fatty acids beta-oxidation | 36 | 19 | 8 | 0.7205 | P96 |
| Vitamin D3 (cholecalciferol) metabolism | 16 | 8 | 2 | 0.74425 | P97 |
| Carnitine shuttle | 72 | 19 | 6 | 0.84246 | P98 |
| Bile acid biosynthesis | 82 | 44 | 20 | 0.87327 | P99 |
| Arachidonic acid metabolism | 95 | 76 | 36 | 0.95202 | P100 |
| 3-oxo-10R-octadecatrienoate beta-oxidation | 27 | 1 | 1 | 1 | P101 |
| Glycosphingolipid biosynthesis - lactoseries | 14 | 3 | 1 | 1 | P102 |
| Blood Group Biosynthesis | 44 | 3 | 1 | 1 | P103 |
| Electron transport chain | 7 | 1 | 1 | 1 | P104 |
| Glycosphingolipid biosynthesis - neolactoseries | 16 | 3 | 1 | 1 | P105 |
| Dynorphin metabolism | 8 | 3 | 1 | 1 | P106 |
| O-Glycan biosynthesis | 16 | 2 | 1 | 1 | P107 |
| Proteoglycan biosynthesis | 27 | 3 | 1 | 1 | P108 |
| Vitamin K metabolism | 3 | 1 | 1 | 1 | P109 |
| Dimethyl-branched-chain fatty acid mitochondrial beta-oxidation | 19 | 4 | 1 | 1 | P110 |

^a^Pathway total indicates the overall number of metabolites that are included in a specific pathway; ^b^Hits.total indicates the number of measured signals that are matched (m/z error<3 ppm) with the metabolites included in the pathway; ^c^Hits.sig indicates the number of matched signals that were significantly changed between phenotypic groups; ^d^ Gamma is an adjusted Fisher’s p-value (null distribution) calculated after permutations to determine the significance of the enriched pathway in Mummichog/ Metaboanalyst (Li et al., 2013; Chong et al., 2018); ^e^Pathways Number listed in Table S3 corresponds to Figure 2 in the main text.

References

Beckonert, O., Keun, H.C., Ebbels, T.M.D., Bundy, J., Holmes, E., Lindon, J.C., et al. (2007). Metabolic profiling, metabolomic and metabonomic procedures for NMR spectroscopy of urine, plasma, serum and tissue extracts. *Nature Protocols* 2(11)**,** 2692-2703. doi: 10.1038/nprot.2007.376.

Broadhurst, D., Goodacre, R., Reinke, S.N., Kuligowski, J., Wilson, I.D., Lewis, M.R., et al. (2018). Guidelines and considerations for the use of system suitability and quality control samples in mass spectrometry assays applied in untargeted clinical metabolomic studies. *Metabolomics* 14(6)**,** 72. doi: 10.1007/s11306-018-1367-3.

Chong, J., Soufan, O., Li, C., Caraus, I., Li, S., Bourque, G., et al. (2018). MetaboAnalyst 4.0: towards more transparent and integrative metabolomics analysis. *Nucleic Acids Res* 46(W1)**,** W486-W494. doi: 10.1093/nar/gky310.

Li, S., Park, Y., Duraisingham, S., Strobel, F.H., Khan, N., Soltow, Q.A., et al. (2013). Predicting network activity from high throughput metabolomics. *PLoS Comput Biol* 9(7)**,** e1003123. doi: 10.1371/journal.pcbi.1003123.

Loeser, R.F., Pathmasiri, W., Sumner, S.J., McRitchie, S., Beavers, D., Saxena, P., et al. (2016). Association of urinary metabolites with radiographic progression of knee osteoarthritis in overweight and obese adults: an exploratory study. *Osteoarthritis Cartilage* 24(8)**,** 1479-1486. doi: 10.1016/j.joca.2016.03.011.

Want, E.J., Wilson, I.D., Gika, H., Theodoridis, G., Plumb, R.S., Shockcor, J., et al. (2010). Global metabolic profiling procedures for urine using UPLC–MS. *Nature Protocols* 5(6)**,** 1005-1018. doi: 10.1038/nprot.2010.50.

Weljie, A.M., Newton, J., Mercier, P., Carlson, E., and Slupsky, C.M. (2006). Targeted profiling: quantitative analysis of 1H NMR metabolomics data. *Anal Chem* 78(13)**,** 4430-4442. doi: 10.1021/ac060209g.
